# Spectrotemporal signatures of driving- and modulatory circuits across cortical and subcortical networks

**DOI:** 10.3389/fnhum.2026.1868940

**Published:** 2026-08-12

**Authors:** M. N. O’Connell, A. Barczak, C. A. Mackey, T. McGinnis, K. Mackin, J. F. Smiley, C. Bleiwas, P. Lakatos, C. E. Schroeder

**Affiliations:** 1 Division of Translational Neuroscience, Center for Biomedical Imaging and Neuromodulation, Nathan S. Kline Institute for Psychiatric Research, Orangeburg, NY, United States; 2 Department of Psychiatry, New York University School of Medicine, New York, NY, United States; 3 Department of Neurological Surgery, Columbia University College of Physicians and Surgeons, New York, NY, United States; 4 Department of Psychiatry, Columbia University College of Physicians and Surgeons, New York, NY, United States

**Keywords:** driving vs. modulatory pathways, entrainment, eye movement, non-human primate, thalamocortical circuits

## Abstract

**Introduction:**

Sensory processing depends on interactions between neural circuits that convey and regulate information across cortical and subcortical networks. Classical frameworks distinguish driving inputs, which transmit sensory content via suprathreshold activation, from modulatory inputs, which alter neuronal excitability without directly eliciting spiking. However, physiological signatures of these circuit types that generalize widely across distributed brain regions remain unclear.

**Methods:**

We functionally differentiated driving and modulatory circuits in the awake macaque brain by jointly quantifying suprathreshold multiunit activity (MUA) and oscillatory phase coherence (inter-trial coherence, ITC) across eight cortical and thalamic structures during auditory, visual, and eye-movement conditions.

**Results:**

Preferred sensory stimuli elicited broadband ITC increases accompanied by robust MUA, yielding relatively uniform spectral distributions across adjacent frequency bands, consistent with driving inputs. In contrast, non-preferred sensory and eye-movement related events produced narrowband, frequency-specific ITC modulation without concurrent firing, characterized by dominant peaks at stimulation or event rates, consistent with modulatory inputs. These two response types were observed within individual regions, revealing two separable modes of neural activity.

**Discussion:**

This narrowband ITC modulation is indicative of coordinated phase alignment, capable of dynamically regulating information transfer mediated by driving inputs across thalamocortical circuits. These findings identify distinct spectrotemporal signatures of driving and modulatory circuit properties across cortical and thalamic levels and demonstrate that subthreshold oscillatory modulation is a widespread phenomenon reflecting cross-modal and saccadic-related influences on neural excitability.

## Introduction

Sensory processing relies on the coordinated activity of distributed neural circuits that convey and transform information from the periphery to cortex. Within this hierarchy, connections between regions can differ profoundly in their effect on target neurons. According to the influential framework first proposed by Sherman and Guillery (1998) and Jones (1998), “driving” inputs transmit the main informative content of a signal, generating suprathreshold activation and shaping receptive field properties, whereas “modulatory” inputs primarily adjust the responsiveness of target neurons without directly driving spiking (Jones, 2001; Lakatos et al., 2007; Schroeder and Lakatos, 2009). These circuit types are not restricted to a single hierarchical level; both cortical and thalamic structures can receive mixtures of driving and modulatory inputs that together determine how sensory events are encoded and integrated (Barczak et al., 2023).

The distinction between driving and modulatory circuits is measurable *in vivo* due to their specific physiological signatures. Driving inputs are typically associated with large, temporally precise synaptic currents that produce clear evoked responses in both local field potentials (LFPs) and multiunit activity (MUA). In contrast, modulatory inputs often produce subthreshold changes in membrane potential through the phase resetting of ongoing neural oscillations (which reflect fluctuations of excitability in neuronal ensembles) (Lakatos et al., 2007; Lakatos et al., 2009, 2020; O’Connell et al., 2020; Shah et al., 2004). Thus, modulatory inputs regulate firing probabilities rather than causing obvious changes in the firing rates of neuron ensembles. Such subthreshold effects are particularly evident in studies utilizing inter-trial coherence (ITC), which indexes the phase alignment of neural oscillations across repeated events. Whereas broadband ITC increases often accompany stimulus-evoked firing, narrowband or low-frequency ITC modulations can reflect subtle entrainment of neuronal excitability - signatures of modulatory influence (Barczak et al., 2019; Obleser and Kayser, 2019; Schroeder and Lakatos, 2009).

Oscillatory modulation plays a critical role in shaping how the brain synchronizes sensory processing across space and time. Rhythmic inputs from auditory or visual streams, for example, can entrain low-frequency oscillations in cortical areas, aligning the phase of neuronal excitability with expected stimulus timing (Besle et al., 2011; Haegens and Zion Golumbic, 2018; Lakatos et al., 2008; Schroeder and Lakatos, 2009). Through this mechanism, modulatory inputs can enhance or decrease the effectiveness of driving signals if they arrive at the optimal or suboptimal oscillatory phase, respectively. Therefore, the functional distinction between driving and modulatory circuits is complemented by a dynamic interaction: modulatory entrainment establishes temporal windows of high or low excitability, while driving inputs convey detailed sensory content within those windows.

Accumulating evidence indicates that sensory pathways are inherently multisensory (Cappe et al., 2009; Ghazanfar and Schroeder, 2006) and are further influenced by internally generated motor signals. Inputs from non-preferred modalities or motor systems have been found to act through modulatory pathways, shaping oscillatory phase and gain rather than directly driving activity. For instance, visual and auditory cortices can exhibit phase resetting in response to stimuli outside their dominant modality (Kayser et al., 2008; Lakatos et al., 2007; Lakatos et al., 2009; Mégevand et al., 2020; Naue et al., 2011; Plass et al., 2019; Romei et al., 2012), and both can be entrained by saccadic eye movements that predictively modulate sensory responsiveness (Barczak et al., 2019; Ito et al., 2013; Leszczynski et al., 2023; O’Connell et al., 2020). These findings suggest that motor-related and cross-modal signals operate primarily via modulatory circuits that influence the timing and excitability of sensory networks rather than transmitting content-rich driving input (with some exceptions: Brosch et al., 2005; Mackey et al., 2024).

The concept of driving versus modulatory circuits is reasonably posited to be a general property of the brain, though its physiological expression across a wide variety of cortical and subcortical areas remains incompletely characterized. Many prior studies have focused on single structures, which makes it difficult to assess how driving and modulatory mechanisms operate in parallel across distributed nodes within sensory hierarchies. In the thalamus, for example, primary relay nuclei such as the medial and lateral geniculate bodies (MGB, LGN) receive well-characterized driving inputs from peripheral sensory organs but also receive modulatory inputs from other subcortical structures, plus feedback from cortex (Barczak et al., 2023; Sherman and Guillery, 2002, 2011). Similarly, cortical sensory areas integrate both bottom-up driving and modulatory inputs (Jones, 1998, 2001), and top-down modulatory influences that shape oscillatory dynamics (Gilbert and Li, 2013; Helfrich and Knight, 2016). Determining if and how these functional distinctions - driving versus modulatory - are reflected in the temporal and spectral characteristics of neuronal responses across regions is critical for understanding how the brain coordinates perception across modalities and hierarchical levels.

Here, we sought to identify physiological signatures of driving and modulatory circuits across auditory, visual, motor and multisensory structures in the awake macaque brain. By simultaneously recording local field potentials and multiunit activity in multiple cortical and thalamic regions, we compared neural responses within these regions to sensory stimuli of preferred and non-preferred modalities, and internally generated saccadic events. We reasoned that driving inputs would elicit strong suprathreshold activation, which would be expressed as a broadband response in the field potential, accompanied by MUA, and that modulatory inputs would primarily induce narrowband oscillatory phase alignment without significant population spiking. By examining these response patterns across multiple structures, we aimed to characterize how driving and modulatory signals are distributed within and across thalamocortical networks. We further hypothesized that modulatory input regulates the phase relationships of oscillatory activity across regions, thereby dynamically facilitating or impeding the transfer of sensory information through driving pathways. In this view, modulatory circuits do not merely synchronize local excitability but align, or misalign, the phases of distributed networks to control when and where driving inputs are most effective. By jointly quantifying suprathreshold and subthreshold responses across auditory and visual thalamic nuclei and cortical areas, our study provides new insights into the functional organization of driving and modulatory circuits in the awake primate brain. Understanding this balance is critical for explaining how neural systems integrate multisensory inputs, including auditory, visual, and motor-related signals, generate predictions about upcoming input, and support coherent perception in dynamic environments.

## Materials and methods

### Subjects

In the present study, eye position and electrophysiological data were recorded from 1 male and 5 female macaques (*Macaca mulatta*) weighing 4.5–10 kg, who had been prepared surgically for chronic awake electrophysiological recordings. There were 207 recording sites distributed across 137 experimental sessions (38, 46, 13, 40, 14 and 56 sites from 19, 32, 9, 30, 10 and 37 sessions with macaques B, G, M, R, T and U respectively). Prior to surgery, each animal was adapted to a custom fitted primate chair and to the recording chamber. All procedures were approved in advance by the Institutional Animal Care and Use Committee of the Nathan Kline Institute.

### Surgery

Preparation of subjects for chronic awake intracortical recording was performed using aseptic techniques, under general anesthesia, as described previously (Schroeder et al., 1998). The tissue overlying the calvarium was resected and appropriate portions of the cranium were removed. The neocortex and overlying dura were left intact. To provide access to the brain and to promote an orderly pattern of sampling across the surface of the auditory areas, Polyetheretherketone (PEEK) recording chambers (Rogue Research Inc.) were positioned normal to the cortical surface of the superior temporal plane for orthogonal penetration of auditory cortex and thalamic structures beneath, as determined by pre-implant MRI. Together with a PEEK headpost (to permit painless head restraint), they were secured to the skull with ceramic screws and embedded in dental acrylic. We used MRI compatible materials to allow for post implant imaging. A recovery time of 6 weeks was allowed before we resumed behavioral training and began data collection.

### Electrophysiology

**Figure 1:**
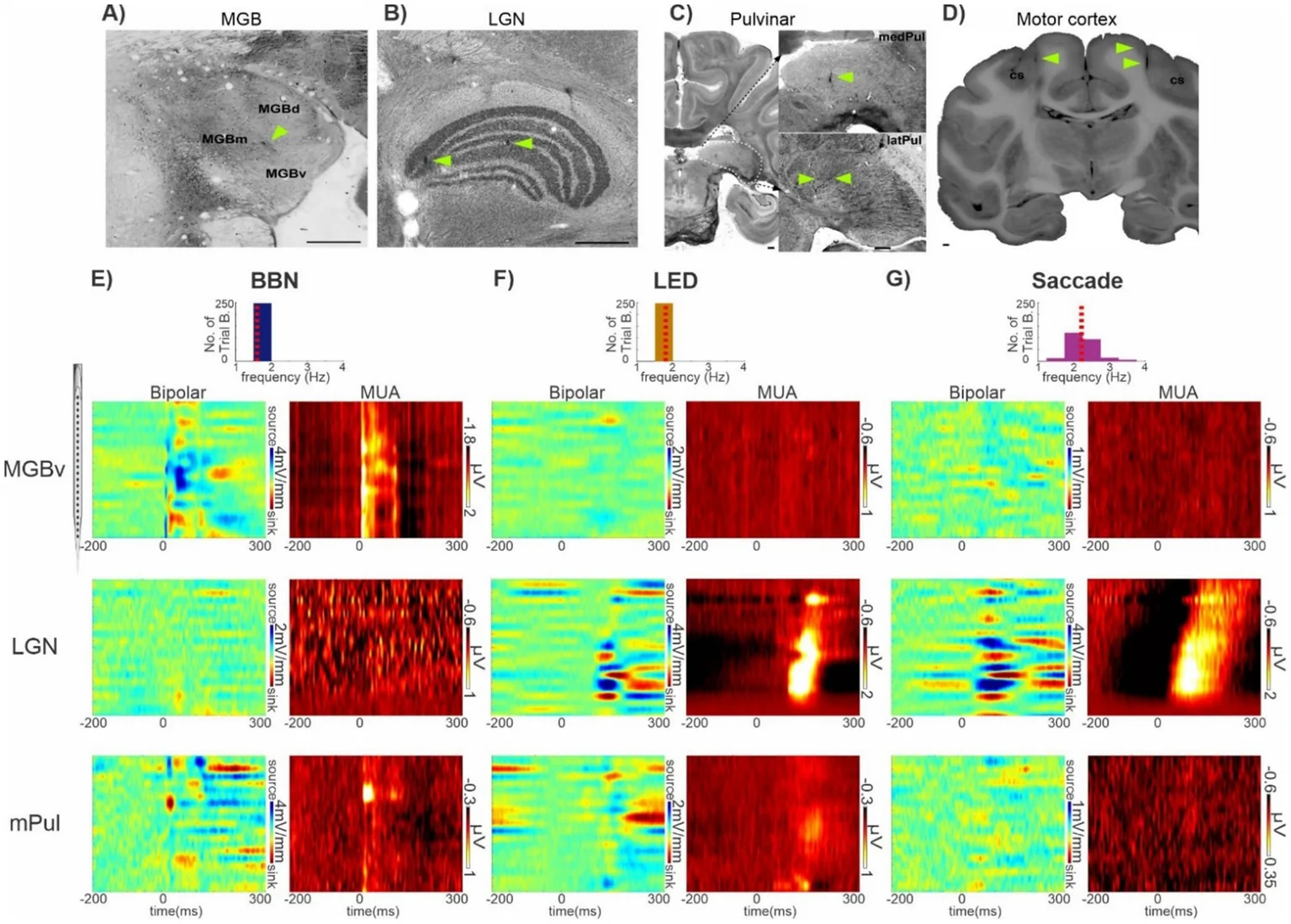
Representative MUA and bipolar field potential profiles associated with preferred and nonpreferred modalities in subcortical structures. **(A–D)** Histological verification of recording sites. **(A)** Calbindin staining of MGB (dorsal, medial, and ventral subdivisions). **(B)** Nissl-stained section of LGN. **(C)** Nissl-stained sections of pulvinar, with higher magnification insets showing electrodes in medial and lateral subnuclei. **(D)** Block-face image taken during brain sectioning, showing electrodes in motor cortex just medial to the central sulcus (cs). Yellow arrows indicate electrode tracks. **(A–C)** are oriented with the medial brain to the left. Scale bars = 1 mm. **(E–G)** Representative bipolar field potential and multiunit activity (MUA) profiles from MGBv (top), LGN (middle), and mPul (bottom), along with corresponding event rate distributions (top panels; red dotted lines indicate median rates). **(E)** Responses to rhythmic broadband noise (BBN; median rate 1.6 Hz). Schematics illustrate linear array electrode placement. **(F)** Responses to rhythmic LED flashes (median rate 1.8 Hz) recorded from the same sites as in **E**. **(G)** Responses aligned to saccade onset during resting state (median rate 2.2 Hz), reflecting quasi-rhythmic motor sampling. Note that different amplitude scales were applied across color plots.

During the experiments, animals sat in a primate chair in a dim, isolated, electrically shielded, sound-attenuated chamber with head fixed in position, and were monitored with infrared cameras. Neuroelectric activity was obtained using linear array multi-contact electrodes (23 contacts, 100 μm intercontact spacing, Plexon Inc.) from 66 A1 (20, 10, 6, 16, 7 and 7 sites in macaques B, G, M, R, T and U respectively), 31 secondary auditory cortex (Belt), (6, 11, 2, 4, 2 and 6 sites in macaques B, G, M, R, T and U respectively), 16 ventral division of the medial geniculate body (2, 5, 1 and 8 sites in macaques G, R, T and U respectively), 34 nonlemniscal medial geniculate body (4, 9, 1, 8, 3 and 9 sites in macaques B, G, M, R, T and U respectively), 11 lateral geniculate nucleus (1, 2, 4 and 4 sites in macaques B, G, R and U respectively), 21 medial pulvinar (8, 1,1, 1 and 10 sites in macaques G, M, R, T and U respectively), 14 pulvinar (2, 4, 2, 1 and 5 sites in macaques B, G, M, R, and U respectively) and 14 motor cortex (5, 1, 1 and 7 sites in macaques B, M, R and U respectively) sites. Subsets of this data from areas A1, pulvinar and medial geniculate body have been used in previous publications from our lab (Barczak et al., 2018; Lakatos et al., 2007, 2013, 2016, 2020; O’Connell et al., 2015, 2020, 2011, 2014). The multielectrodes were inserted acutely through guide tube grid inserts, lowered through the dura into the brain, and positioned such that the electrode channels would span as much of a sub-cortical nucleus as 2.2 mm would allow (see section below titled “Identification of Recording Structures” and Figure 1), and all layers of a cortical region. Final electrode position was guided by creating and inspecting current source density (CSD) and multiunit (MUA) response profiles to binaural broadband noise bursts and strobe flashes. Neuroelectric signals were impedance matched with a pre-amplifier (10x gain, bandpass dc-10 kHz) situated on the electrode, and after further amplification (500x) they were recorded continuously with a 0.01–8,000 Hz bandpass digitizer with a sampling rate of 44 kHz and precision of 12-bits using the Alpha Omega SnR system. The signal was split into the field potential (0.1–300 Hz) and MUA (300–5,000 Hz) range by zero phase shift digital filtering. MUA data was also rectified in order to improve the estimation of firing of the local neuronal ensemble (Legatt et al., 1980). One-dimensional current source density (CSD) profiles were calculated from the local field potential profiles using a three-point formula for the calculation of the second spatial derivative of voltage (Freeman and Nicholson, 1975). The advantage of CSD profiles is that they are not affected by volume conduction like the local field potentials (Kajikawa and Schroeder, 2011, 2015), and they also provide a more direct index of the location, direction, and density of the net transmembrane current flow (Mitzdorf, 1985; Schroeder et al., 1998). Bipolar signals were derived by computing the difference between adjacent contacts along each linear electrode array. This spatial differentiation reduces the influence of volume-conducted activity and enhances sensitivity to locally generated signals. Bipolar derivations were used for analyses of subcortical recordings, where laminar current source density estimates are not applicable.

### Eye tracking and saccade detection

Eye position was recorded at 500 Hz using an EyeLink 1,000 Plus system with the animal’s head immobilized. The distance from each animal’s eye to the camera ranged from 23.5–25.5 inches across the 6 NHPs; however, for each individual animal this distance remained consistent across recording sessions. This was ensured by placing the EyeLink camera in the same marked location for each animal, with head immobilization maintained via head holders fixed to custom-fitted chairs that were secured to a fixed frame within the recording booth. Head angles were maintained as consistently as possible across sessions. Saccades were detected using the Engbert and Kliegl (2003) algorithm, which estimates the mean and standard deviation of eye velocity during fixation from the peak of the velocity distribution for each individual session and sets the saccade threshold at mean + *λ* × SD (λ = 6). Because both the raw signal and the threshold are derived from the same session’s data, any session-to-session variations in hardware gain, such as differences in analog amplifier settings or small changes in camera angle, affect the raw velocities and the threshold by the same factor, effectively canceling out. This self-correcting property of the algorithm ensures consistent saccade detection across sessions without requiring formal spatial calibration. Additional detection criteria were applied to minimize noise artifacts and ensure detection of genuine saccades: saccades were required to last at least 8 ms (4 consecutive samples at 500 Hz) with a minimum inter-saccade interval of 120 ms, and only trial blocks containing at least 40 verified saccades were included in analyses. All detected saccades were manually verified and artifacts rejected by two experienced lab members with expertise in saccade detection. This approach to saccade detection has been used previously in our lab (O’Connell et al., 2020). Saccade velocity and duration distributions across all 6 NHPs are provided in Supplementary Figure 1.

### Stimulus presentation

At the beginning of each experimental session, streams of 50 dB SPL broadband noise (BBN) bursts and strobe flashes (Grass Instrument photo stimulator) were presented separately to elicit auditory and/or visual neural responses to locate brain structures and determine final electrode position. Once electrode position was refined, the frequency tuning of each recording site was determined using a “suprathreshold” method (Fu et al., 2004; Schroeder et al., 2001; Steinschneider et al., 1995). The method entailed the presentation of a pseudorandom train of 14 different frequency pure tones ranging from 353.5 Hz to 32 kHz in half octave steps and a BBN burst at 50 dB SPL [duration: 100 ms, rising/falling time: 4 ms, stimulus onset asynchrony (SOA) = 319 ms, *n* = 1,600]. Next, for the collection of the data used for the main analyses in this paper, subjects sat passively in a dim sound attenuated chamber through three conditions. Condition 1 included the presentation of a rhythmic stream of BBN bursts at 50 dB SPL (duration: 100 ms, r/f time: 4 ms, SOA = 624 ms, corresponding to a 1.6 Hz repetition rate). Every BBN stream trial block consisted of approximately 100 BBN bursts and thus lasted just over 1 min. Auditory stimuli were produced using Tucker Davis Technology’s System III coupled with two MF-1 free field speakers which were positioned 12 inches diagonally in front of each subject at ear level. Condition 2 included the presentation of a rhythmic stream of LED flashes which were 25 ms in duration and had an SOA of 562 ms (corresponding to a 1.8 Hz repetition rate). LED intensity was fixed and was not calibrated to individual subject thresholds. Eye position was tracked throughout visual stimulation but was not formally controlled. Each LED stream trial block consisted of approximately 500 LED flashes and thus lasted almost 5 min. The LED was positioned 39 inches directly in front of the subject at eye level. In a subset of experiments (*n* = 33), the LED stream was presented at a theta rate of 6 Hz (SOA of 166 ms). Condition 3 included the absence of any stimuli (resting state condition) during which saccades were detected. The resting state trial block lasted approximately 5 min. There was no behavioral task in any condition and thus no behavior was required of the NHP. Animals were kept alert by interacting with them prior to and after each trial block.

### Identification of recording structures

Consultation of a macaque anatomy atlas with stereotaxic co-ordinates guided the location of each electrode penetration. While our main recording target sites were motor cortex, primary auditory cortex (A1) and the auditory thalamic nucleus [the medial geniculate body (MGB)], we inadvertently but serendipitously recorded from other brain structures. Auditory cortical recording sites were initially identified based on location in the recording chamber, depth from superior surface of the brain and responsiveness to auditory stimuli. Primary (A1) and secondary (Belt) auditory cortical recording sites were separated based on the sharpness of frequency tuning, the inspection of the tonotopic progression across adjacent sites, onset latencies and relative sensitivity to pure tones versus broad-band noise of equivalent intensity (Lakatos et al., 2005a; Merzenich and Brugge, 1973; Rauschecker et al., 1997). Motor cortical recording sites were characterized based on their more anterior position in the recording chamber, their shallow depth from the top of dura, the inversion of local field potentials to auditory stimuli and lack of frequency tuning. Motor cortex recording sites were localized using stereotaxic coordinates (Supplementary Figure 2). Mean AP coordinate was 14.36 ± 1.91 mm (range: +12 to +17 mm) and mean ML coordinate was 14.14 ± 3.18 mm (range: +9 to +19 mm), consistent with primary motor cortex (M1) (Saleem and Logothetis, 2012). No sites were located anterior to AP + 17 mm or medial to ML + 9 mm, confirming that all recordings were within M1 and excluding localization to premotor cortex (PMd) or supplementary motor area (SMA) respectively.

Thalamic and subcortical structures were, at first, determined based on depth from superior surface of the brain and primary responsiveness to either auditory or visual stimuli [e.g., MGB and lateral geniculate nucleus (LGN)]. The classification of recording sites located in the ventral division of MGB (MGBv) as opposed to in the dorsal and ventral subdivisions of the nucleus (MGB) was determined by comparing MUA response onset latencies to BBN and pure tone stimuli, and sharpness of frequency tuning curves. The ability to separately identify lateral pulvinar and LGN recording sites was based on recording depth, onset latencies of MUA responses to visual stimuli and medial-lateral location relative to other identified structures in the recording chamber. Medial pulvinar recording sites were labelled as such due to their recording depth and responsiveness to both auditory and visual stimuli. At the end of each animal’s experimental participation, functional assignment of the recording sites was confirmed histologically (Figures 1A–D) (Lakatos et al., 2020; Barczak et al., 2018; Schroeder et al., 2001).

### Data analysis

**Figure 2:**
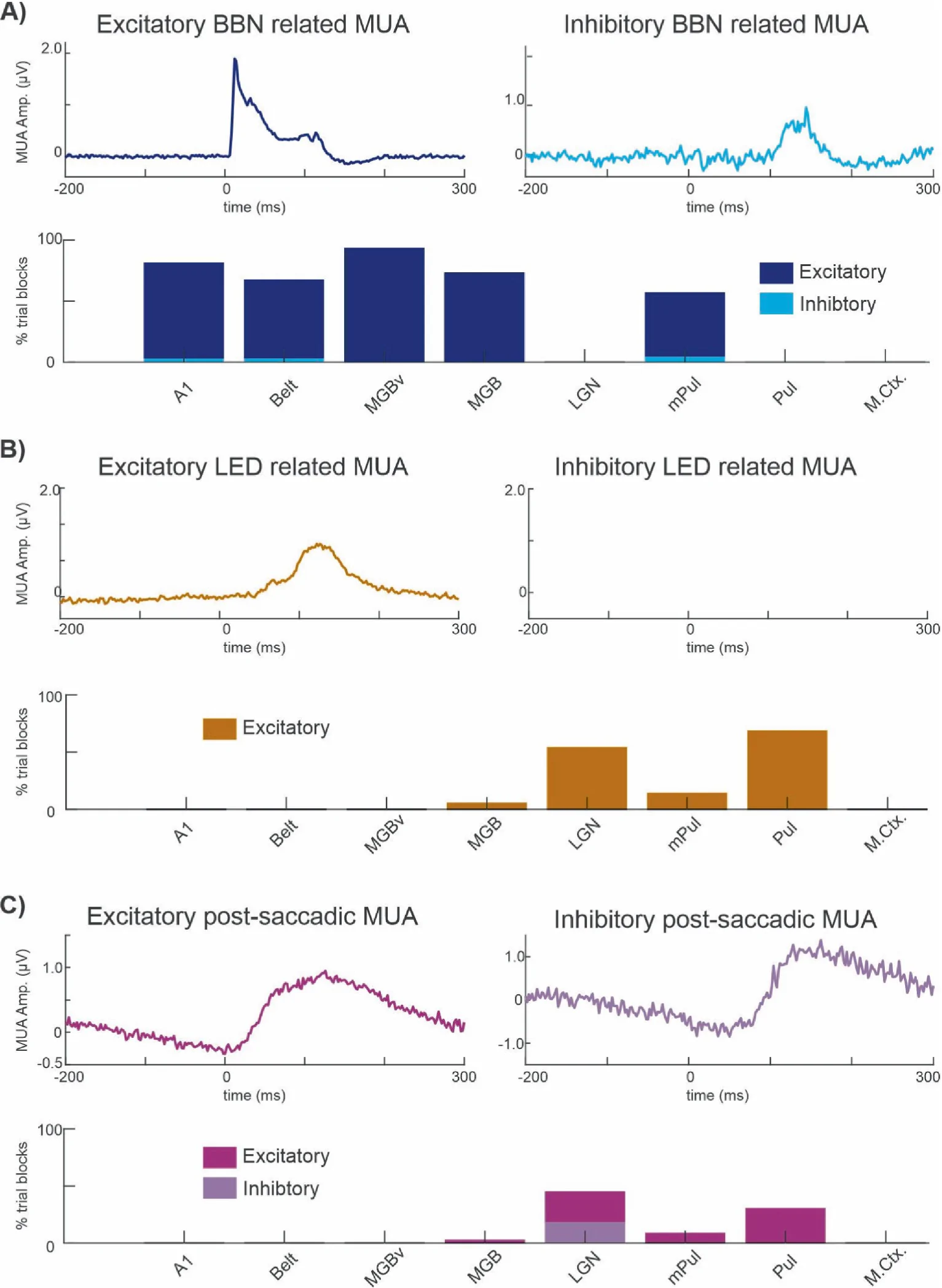
Excitatory and inhibitory suprathreshold MUA responses across 8 cortical and subcortical brain structures. **(A–C)** Average MUA waveforms and corresponding bar graphs summarizing the proportion of recording sites exhibiting significant evoked responses (0–300 ms) across brain regions. Left and right traces show excitatory and inhibitory responses, respectively, color coded by condition. Bar graphs indicate the percentage of sites within each region exhibiting each response type. **(A)** BBN-related responses. Navy traces indicate excitatory responses and cyan traces indicate inhibitory responses. **(B)** LED-related responses. Mustard traces indicate excitatory responses; no inhibitory responses were observed. **(C)** Saccade-related responses. Purple and light purple traces indicate excitatory and inhibitory responses, respectively.

Data were analyzed offline using native and custom-written functions in Matlab (Mathworks, Natick, MA). To identify suprathreshold event-related MUA responses at each electrode contact, we statistically compared the post-event (0–300 ms) activity of “real” MUA epochs - time-locked to the event of interest (e.g., BBN onset) - with that of “control” MUA epochs using a Wilcoxon rank-sum test. Bonferroni correction was applied to account for multiple comparisons across both recording contacts within each structure and timepoints within each analysis window. An evoked response was defined as a period of at least 4 consecutive milliseconds in which MUA activity significantly differed between real and control epochs. Control MUA epochs were generated by randomly selecting timepoints within each trial block to serve as pseudo-event onsets, with the number of random timepoints matched to the number of real event onsets in that block. For each structure, if multiple linear-array contacts exhibited significant evoked MUA responses, the contact whose significant response was closest to event onset (0 ms) - whether excitatory or inhibitory - was selected for display in Figure 2.

To quantify the phase coherence of neuronal oscillatory activity across trials, the instantaneous phase of each single trial signal was extracted from CSD signals for cortical sites and from bipolar derivations for subcortical sites using Morlet wavelet decomposition across 69 frequency scales ranging from 0.7 to 50 Hz. ITC values range from 0 to 1; higher values indicate that the observations (oscillatory phase at a given time-point across trials) are clustered more closely around the mean than lower values (phase distribution is random). To locate significant ITC peaks, we first determined the number of ITC peaks across all electrode contacts spanning a brain structure, for each of the 69 frequencies (0.7–50 Hz) within the post-event time window (0 to 300 ms) using the *findpeaks* function in Matlab. For each trial block, the statistical significance of each ITC peak was evaluated using Rayleigh’s uniformity test. A Bonferroni corrected *p*-value was determined individually for each frequency based on the number of ITC peaks detected across all electrode contacts within a brain structure at that frequency (i.e., *p*-value corrected for multiple comparisons = *α* / number of detected peaks across all electrode contacts at that frequency).

To statistically compare the broadness of significant ITC peak distribution across frequency bands between evoked and modulatory responses, we quantified the cumulative area under the significant ITC peak distribution curve for each trial block using Matlab’s *cumtrapz* function. Frequency band boundaries were predefined, and the cumulative area between each pair of boundaries was extracted to obtain the area under the curve (AUC) for each frequency band. These were then pooled by response type (i.e., evoked vs. modulatory) across all areas and compared using a Kruskal-Wallis test with Bonferroni correction.

To determine the temporal extent of frequency-band-specific differences in significant ITC peak counts across response types within each area, simultaneous 95% confidence bands were estimated using 1,000 bootstraps for each frequency. Binary significance matrices - defined as time–frequency points where the lower 95% confidence limit of one response type (e.g., evoked ITC) exceeded the upper 95% confidence limit of another (e.g., modulatory ITC) - were then collapsed across predefined frequency bands. For each band, significance values were summed across frequencies and converted into a binary time series, where a value of 1 indicated that at least one frequency bin within the band showed a significant difference at that timepoint. This procedure yielded time-resolved horizontal lines, which illustrate when ITC peak counts associated with one response type were significantly greater than those for the other within each frequency band.

To determine the phase alignment of MUA, the instantaneous phase of each single-trial signal was extracted from the mean translaminar/transnuclear MUA at each site using Morlet wavelet decomposition (as above). For BBN and LED streams, which were presented isochronously at 1.6 Hz and 1.8 Hz respectively, the mean MUA phase at the corresponding stimulus frequency was measured at the onset of each stimulus. For saccades, which are aperiodic, the frequency of the peak MUA ITC value within the delta-theta band was identified for each site and used to extract the mean MUA phase of each resting state trial block.

## Results

### Evoked multiunit activity (MUA) responses in cortical and subcortical brain structures related to auditory, visual and saccadic events

Laminar field potential and concomitant multiunit activity (MUA – indexing spiking activity in local neuronal ensembles) profiles were obtained with linear array multicontact electrodes (Figure 1E) from 66 primary auditory cortex (A1), 31 secondary auditory cortex (Belt), 16 ventral division of the medial geniculate body (MGBv), 34 medial geniculate body (nonlemniscal medial and dorsal divisions, grouped together) (MGB), 11 lateral geniculate nucleus (LGN), 21 medial pulvinar (mPul), 14 lateral pulvinar (Pul) and 14 primary motor cortex sites, across 6 awake macaque monkeys (see Figures 1A–D for representative histological examples). Neuroelectric activit was recorded under three conditions: (1) during the presentation of a rhythmic (1.6 Hz) stream of 50 dB, 100 ms duration broadband noise (BBN) bursts (auditory stimulation condition), (2) during the presentation of a rhythmic (1.8 Hz) stream of LED flashes (visual stimulation condition), and (3) in the absence of any stimuli during which saccades were detected (resting state condition). NHPs were seated in a dim, electrically shielded, sound-attenuated chamber and remained alert, but no behavioral task was required in any condition. Eye position was continuously monitored and the NHPs were free to look around anywhere in the room and at their naturally preferred pace. (see Methods for more details.) Since only one trial block of each condition was recorded in each of the recording sites, from this point on we will use the term sites as opposed to trial blocks or recordings. For cortical recordings, CSD profiles were calculated from field potential profiles. CSD is used as an index the location, direction, and density of transmembrane current flow, which is the first-order neuronal response to synaptic input (Nicholson and Freeman, 1975; Schroeder et al., 1998). Since it is uncertain whether the neural tissue in thalamic areas meets the fundamental assumptions required for applying 1D CSD, which are that neuronal tissue is infinite, homogeneous and an isotropic conductive medium (Mitzdorf, 1985; Nicholson and Freeman, 1975), we opted to calculate the bipolar derivation instead for our thalamic recordings. This method still localizes neural activity and, similar to CSD, is less susceptible to contamination by volume conduction (Biagioni et al., 2025; Kajikawa and Schroeder, 2011).

Figure 1E shows example laminar bipolar field potential and MUA profiles to a BBN stream from a representative MGBv site (top), LGN site (middle) and mPul site (bottom). As expected, responses in MGBv are characteristic of a driving or evoked type consistent with its strong ascending projections from the central nucleus of the inferior colliculus. This response is marked by an early (~5 ms post-stimulus), large-amplitude, and sustained increase in MUA across all recording contacts with an accompanying increase in the bipolar field potential signal (Figure 1E, top). In contrast, the LGN site shows no suprathreshold post-stimulus firing or appreciable bipolar response to BBN presentation (Figure 1E, middle). This is not surprising as the LGN is the primary visual nucleus in the thalamus. However, the mPul site, a multisensory thalamic structure, exhibits an intermediate response profile: BBN stimuli evoke an early (~8 ms post-stimulus), transient increase in MUA along with a corresponding bipolar field potential response. However, both signals are lower in amplitude than those observed in MGBv (Figure 1E, bottom, note different amplitude scales).

When we consider responses to visual stimuli (LED flashes) in these same subcortical recording locations as Figures 1E,F, we find that MGBv shows minimal bipolar or MUA activity. (top). Also unsurprisingly, LGN responds with a large amplitude bipolar field potential and a concomitant sustained MUA increase approximately 100 ms post LED flash (Figure 1F, middle). Akin to the response profile that auditory stimuli elicit in MGBv, this is again characteristic of an evoked type response. The mPul site also shows an evoked response, with suprathreshold MUA activity emerging at ~120 ms, accompanied by a lower-amplitude bipolar signal relative to LGN (Figure 1F, bottom, note the different amplitude scales).

In addition to environmental stimuli (i.e., auditory and visual) we also examined whether saccades, an internally generated motor sampling pattern, could result in evoked responses within any of the eight cortical and subcortical brain structures we examined. Unlike the auditory and visual stimuli which were presented completely isochronously, saccades occurred quasi-rhythmically (see inset presentation rate bar graphs at the top of Figures 1E–G). The distribution of median saccade rates across all 207 resting state sites illustrates this and also supports similar findings in numerous human and non-human primate studies (Barczak et al., 2019; Hoffman et al., 2013; Maldonado et al., 2008; Rayner, 1998). The median rate of the individual median saccade rates was 2.2 Hz (median absolute deviation = 0.24 Hz) which is slightly lower than in previous non-human primate studies (Barczak et al., 2019; Berg et al., 2009; Ito et al., 2011), likely due to the lack of a visual task and strict criteria used for saccade detection (see Methods).

Aligned to saccade onset, the MGBv site again shows minimal bipolar or MUA modulation (Figure 1G, top). However, in the representative LGN site (Figure 1G, middle) there is clear MUA suppression prior to and around the time of saccade onset, which is followed by a large amplitude MUA excitation approximately 50 ms after saccade onset. As has been documented previously in the LGN, this suppressive/excitatory MUA pattern is typical of saccadic suppression followed by excitation related to the visual input at saccade end (Lee and Malpeli, 1998; Reppas et al., 2002). The MUA enhancement is accompanied by a large-amplitude bipolar signal and is comparable in magnitude to the LED-evoked response, although occurring at shorter latency. This is possibly due to the fact that saccading even in a dim room provides richer stimulation than a visually weak (low intensity) stimulus like the LED flash, which would result in slow retinal integration and hence longer latency activation. In the mPul site, saccades do not elicit clear suprathreshold MUA responses but produce a low-amplitude bipolar modulation emerging ~60 ms after saccade onset (Figure 1G, bottom).

Next, we wanted to quantify the suprathreshold MUA changes seen in the representative sites in Figure 1, across all sites recorded in the eight brain regions. To do this we statistically tested whether MUA recorded at any linear array electrode contact within a structure significantly differed in amplitude from a “control” MUA signal, created from random segments of the “real” MUA recorded at the same electrode contact, using Wilcoxon rank sum tests (*p* < 0.05 with Bonferroni correction) for at least 4 ms (see Methods), within a traditional post-stimulus/post-saccadic timeframe (0 to 300 ms). If multiple recording contacts within a structure showed significant evoked MUA responses, we selected the contact with the earliest significant response relative to stimulus onset (0 ms), whether excitatory or inhibitory, for display in Figure 2. Population mean MUA time courses for each area and stimulus condition are shown in Supplementary Figure 3.

During the post-stimulus timeframe, we found that many recording locations exhibited BBN-related MUA responses that were significantly larger than their corresponding “control” MUA, which we termed excitatory evoked responses. The top left waveform in Figure 2A displays the grand average of MUA recorded on those recording contacts which displayed excitatory BBN related responses (122/207 or 59% of BBN sites), across all 8 brain structures. A few of the recording locations displayed BBN related MUA responses that were significantly suppressed compared to their matching “control” MUA signal, these we termed inhibitory evoked responses. The waveform in Figure 2A (right) shows the grand average of BBN related inhibitory responses (4/207 or 1.9% of BBN sites). The fact that an obvious post-stimulus MUA suppression is not visible in the grand average is most likely due to the differing latencies of the inhibitory responses across the 4 sites, and thus the responses cancel each other out when averaged. However, the latency of the BBN related off responses are coincident across the four sites, and so there is a visible MUA enhancement right after 100 ms. The bar graph in Figure 2A displays the distribution of excitatory/inhibitory evoked responses across the 8 subcortical and cortical brain structures. The regional distribution of these responses for BBN stimuli is summarized in columns 1 and 2 of Table 1.

**Table 1:** Distribution of excitatory and inhibitory evoked MUA responses across cortical and thalamic regions for auditory (BBN), visual (LED), and saccade (SAC) conditions.

Region	BBN		LED		SAC
	Excit. %	Inhib. %	Excit. %	Excit. %	Inhib. %
A1	78	3	0	0	0
Belt	65	3.2	0	0	0
MGBv	94	0	0	0	0
MGB	73.5	0	5.8	3	0
LGN	0	0	54.5	27	18
mPul	50	4.5	18	9	0
Pul	8	0	69	31	0
Motor cortex	0	0	0	0	0

Values indicate the percentage of recording sites within each region exhibiting excitatory (Excit.) or inhibitory (Inhib.) responses.

During the post-LED flash timeframe (0–300 ms), we found that 21/207 or 10.1% of LED sites showed excitatory MUA evoked responses (see waveform plots in Figure 2B). None of the brain structures we recorded from exhibited significant inhibitory responses to LED flashes. The distribution of brain structures showing excitatory evoked LED flash related MUA responses is displayed in the mustard bar graph (Figure 2B), the corresponding values are provided in column 3 of Table 1.

When we statistically tested saccade related MUA responses, we found that during the post-saccadic timeframe 10/207 or 4.8% of resting state sites resulted in excitatory saccade related evoked MUA responses (Figure 2C, left waveform), while only 2/207 or 0.96% of resting state sites resulted in evoked inhibitory responses (Figure 2C, right waveform). Figure 2C (bar graph) shows the distribution of excitatory and inhibitory evoked MUA responses to saccades across brain structures; the corresponding values are provided in columns 4 and 5 of Table 1. As the brain structures which showed the majority of post-saccadic saccade related excitatory responses are considered traditionally visual or multisensory areas (e.g., LGN and Pul), it is likely that the excitation seen is due to the volley of visual information that enters the brain at saccade end rather than being purely motor related.

### Spectral properties of responses related to auditory, visual and saccadic events

**Figure 3:**
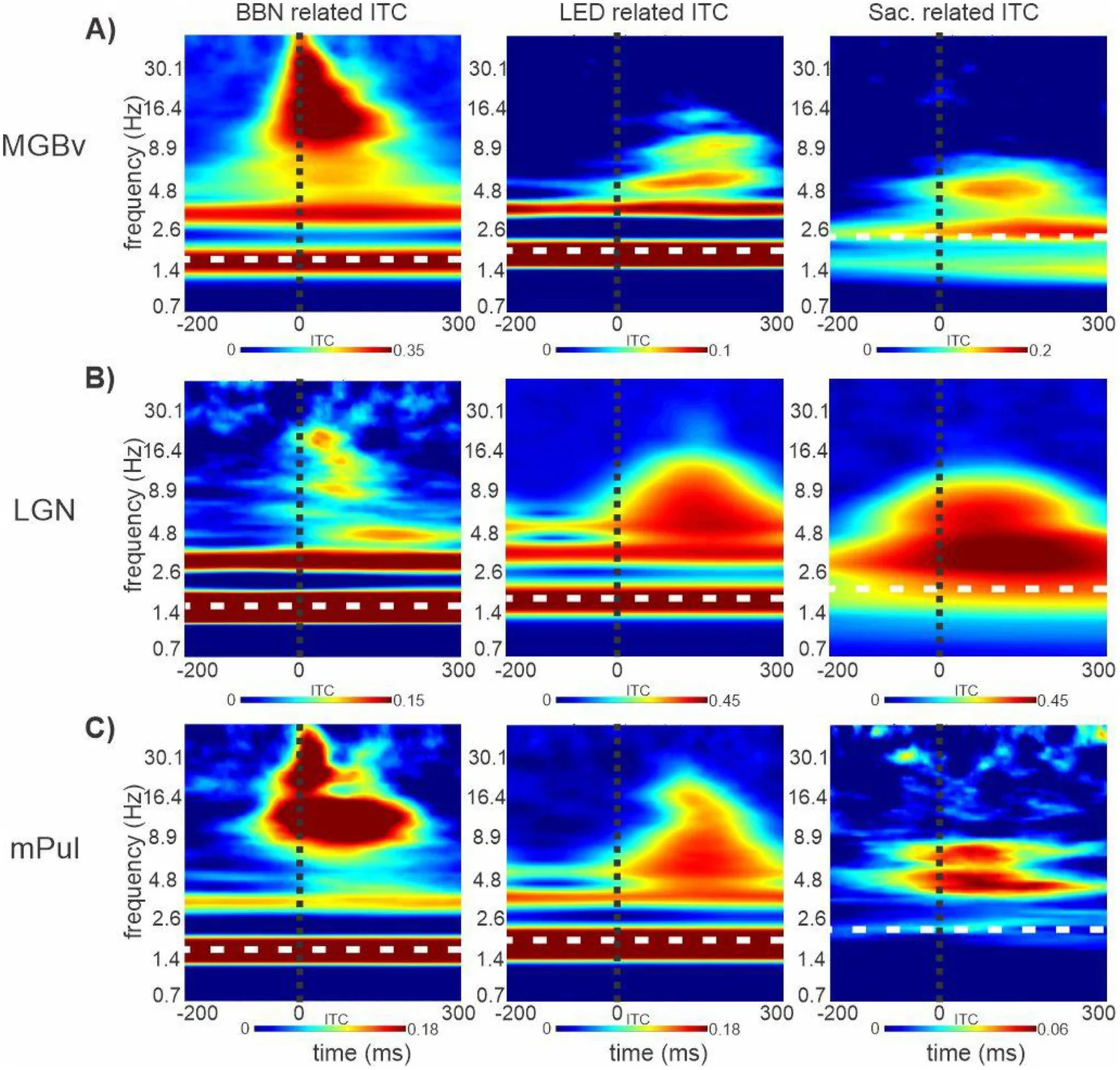
Spectrotemporal properties of event-related responses to preferred and non-preferred modality events in subcortical structures. **(A–C)** Time-frequency plots of bipolar inter-trial coherence (ITC) for representative recordings from MGB **(A)**, LGN **(B)**, and medial pulvinar (mPul; **C**), corresponding to the sites shown in Figure 1. White dotted horizontal lines indicate the stimulus or event rates [BBN: 1.6 Hz; LED: 1.8 Hz; saccades: Median rate for each block (**A**: 2.4 Hz; **B**: 2.17 Hz; **C**: 2.32 Hz)], and black vertical lines mark time 0 ms.

In the past, a common approach to ascertain whether a brain region was involved in the processing of a certain modality was to test if a stimulus of that modality elicited a suprathreshold evoked response (i.e., neuronal firing) in that area (Hubel and Wiesel, 1962; Mountcastle, 1957; Sherman and Guillery, 2002), not unlike the analysis conducted above. In more recent years it has become clear that stimulus related inputs do not always result in obvious “output like” changes in firing rates of neuronal ensembles. Instead, these inputs can adjust the excitability phase of subthreshold ongoing neuronal oscillatory activity, giving rise to modulatory responses. In sensory cortices, this response type has been shown to be most prevalent for non-preferred modality stimuli (Lakatos et al., 2007, 2008) and saccades (Barczak et al., 2019; O’Connell et al., 2020). Our next set of analyses focused on identifying the signatures of modulatory responses across the 8 brain structures to the different modality events. To do this, we investigated the phase coherence of oscillatory activity across sites (indexed by intertrial coherence - ITC) (Lakatos et al., 2005b, 2007). The value of ITC will be 1 in the extreme case where the oscillatory excitability phase is the same in each trial, and it will be 0 if the oscillatory excitability phase across trials is random. Time-frequency plots in Figure 3 display average (across all electrode contacts within a structure) auditory, visual and saccade related ITC values derived from the bipolar signal, from the same three representative subcortical structures shown in Figure 1. Population-mean ITC spectrotemporal heatmaps for each brain area and stimulus condition are shown in Supplementary Figure 4. Within MGBv sites, ITC in response to BBN stimuli is marked by high values across nearly all frequencies within the conventional oscillatory frequency bands (delta through gamma, 1-50 Hz), consistent with an evoked-type waveform (Lakatos et al., 2007, 2009). In contrast, LED flashes and saccades elicit lower overall ITC values, confined to specific frequencies within select oscillatory frequency bands - such as delta, theta, and alpha for LED, and delta and theta for saccades (Figure 3A, middle and right). This phenomenon of ITC values confined to restricted frequencies accompanied by no detectable.

MUA firing (see Figures 1F,G), is thought to be characteristic of a modulatory response (e.g., Lakatos et al., 2007, 2009). Note that in the delta frequency range, ITC values are higher at the rate of stimuli presentation or saccades (marked by white dotted lines). This is due to the entrainment or alignment of the neuronal oscillatory fluctuations (with a similar excitability phase) to the rhythmically presented stimuli and quasi-rhythmic saccades.

In the representative LGN site, LED flashes and saccades resulted in high ITC values broadly across delta through alpha frequency bands (Figure 3B), indicating the evoked responses seen in Figure 1. Since there are no known auditory inputs to LGN and no observed evoked response (Figure 1E), the reduced ITC values peaking at specific frequencies within the delta (approximately 1.6 Hz - implying entrainment), theta, alpha and beta frequency bands in response to the BBN stimuli is suggestive of a modulatory response.

In the multisensory mPul site, although ITC values were lower overall, BBN and LED flashes lead to significant ITC values at the delta stimulation rates, indictive of neural entrainment. This was coupled with broadband ITC values across a range of frequency bands, consistent with the evoked responses seen in Figures 1E,F, 3C. In contrast, saccadic events produced much lower ITC values, which were almost exclusively restricted to the theta frequency band.

**Figure 4:**
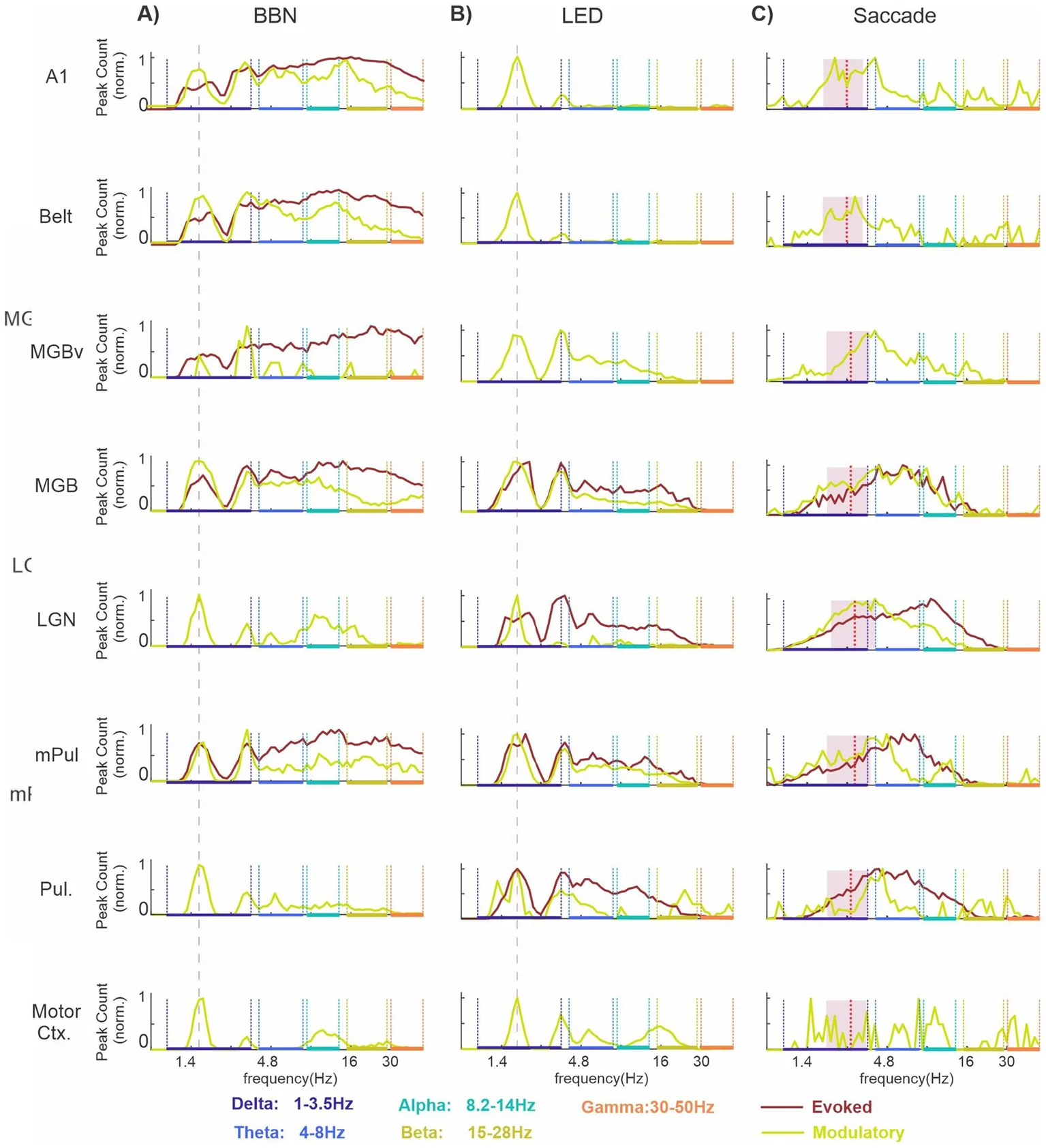
Frequency distribution of significant CSD and bipolar ITC peaks related to multi-modal events across brain structures. **(A–C)** Frequency distributions of significant ITC peaks (1–50 Hz, 0–300 ms) across cortical and subcortical regions. Traces show evoked responses (brown) and non-evoked (modulatory) responses (green). Colored bars on the x-axis indicate the five frequency bands used for subsequent analyses. **(A)** BBN-related responses; the red vertical line indicates the BBN presentation rate (1.6 Hz). **(B)** LED-related responses, shown with the same format as in **A**. **(C)** Saccade-related responses; red vertical lines indicate median saccade rates for each structure, and mauve shading denotes +/− median absolute deviation.

To determine which sites exhibited a purely modulatory (signified by clustering of significant ITC values at specific frequencies) versus an evoked (indicated by significant broadband ITC across multiple frequency bands paired with a suprathreshold MUA) response, we first searched for significant ITC peaks. ITC peaks were evaluated using Rayleigh’s uniformity test (*p* < 0.05 with Bonferroni correction), across individual recording contacts within the post-event time windows (i.e., 0 to 300 ms) and across each frequency band (1-50 Hz) during all sites (see Methods). Traces in Figure 4A show the frequency distribution of significant BBN related ITC peaks (normalized to the peak of each trace for visualization purposes) obtained from BBN sites which resulted in evoked MUA responses (brown traces) vs. non-evoked (green traces), across the 8 brain structures. Counts of significant BBN-related ITC peaks for each region are shown in the first two columns of Table 2. A prominent ITC peak at the BBN presentation rate of 1.6 Hz (red dotted line) was observed across all brain regions, particularly for non-evoked sites, indicating strong entrainment of local delta oscillations to a consistent phase by the BBN stream. In regions where BBN evoked robust MUA responses - such as A1, Belt, and MGBv (see Figure 2A), ITC responses also showed a broad distribution, with a “tail” of an equally large number of ITC peaks across the conventional oscillatory frequency bands. In contrast, sites without evoked responses (e.g., in the LGN and motor cortex) displayed ITC peaks that were more narrowly restricted to distinct frequency bands. This pattern suggests that BBN stimuli organize ongoing oscillatory activity on distinct timescales, as evidenced by the frequency-specific distribution of ITC peaks.

**Table 2:** Number of significant ITC peaks across brain regions and stimulus conditions.

Region	BBN		LED		SAC		BBN	LED	SAC
	Evoked	Modul.	Evoked	Modul.	Evoked	Modul.	Total	Total	Total
A1	74,231	4,961		10,343		1,034	79,192	10,343	1,034
Belt	24,452	5,410		5,082		873	29,862	5,082	873
MGBv	8,930	44		4,735		1,670	8,974	4,735	1,670
MGB	12,823	2,715	2,362	13,436	672	4,142	15,538	15,798	4,814
LGN		652	11,092	296	6,696	2,515	652	11,388	9,211
mPul	11,031	1,663	3,891	9,766	1,433	1,408	12,694	13,657	2,841
Pul		1,599	9,761	960	3,231	490	1,599	10,721	3,721
Motor Crtx		654		2,449		80	654	2,449	80

Peak counts are shown for broadband noise (BBN), LED, and saccade (SAC) conditions, separated into evoked and modulatory (Modul.) responses. Blanks indicate that no significant ITC peaks were detected.

Although the total number of significant LED-related ITC peaks was lower than for BBN stimuli, the overall pattern across regions was similar (see Table 2, columns 3–4). Every sampled brain structure showed a substantial number of significant ITC peaks at the rate of LED flash presentation (1.8 Hz; red line), indicating entrainment. This effect was particularly pronounced in areas in which non-evoked responses to LED flashes predominated (e.g., green traces Figure 4B). Additionally, in recording sites that exhibited primarily evoked responses to LED stimuli - such as the LGN and Pul (e.g., brown traces in Figure 4B) - significant ITC peaks were broadly distributed across adjacent frequency bands, ranging from delta through beta. In the case of non-evoked responses, ITC peaks were typically concentrated within the delta band near the stimulation rate (e.g., A1 and Belt), or clustered around distinct frequencies within specific bands, such as in the Motor Cortex. Unlike BBN stimuli, LED flashes did not elicit many significant ITC peaks in the gamma frequency band. Notably, a prominent number of ITC peaks related to both BBN and LED flash stimuli were observed around 3 Hz across all recording sites. This likely reflects a harmonic relationship, as 3.2 Hz and 3.6 Hz are shared harmonic of the BBN and LED presentation rates (1.6 Hz and 1.8 Hz, respectively).

Saccade-related responses yielded the lowest number of significant ITC peaks across regions; detailed counts are provided in the fifth and sixth columns of Table 2. Surprisingly, the ITC peak distribution traces shown in Figure 4C appear relatively ‘flatter,’ lacking distinct, sharply defined peaks. For non-evoked responses, ITC peaks are primarily concentrated in the high delta to lower theta frequency range. In contrast, saccade-related evoked responses are biased toward higher theta to alpha frequencies, with ITC peaks predominantly concentrated within these bands. A clear distinction between the ITC frequency distributions for sensory stimuli and those related to saccades is the absence of a prominent peak at the median saccade rate (indicated by red lines), which would typically suggest entrainment to a rhythmic saccadic pattern. One possible explanation is that our strict criteria for saccade detection (see Methods) may have led to the omission of some saccades, resulting in an underestimated median rate. Alternatively, if all saccades were accurately detected, the observed peak around 4 Hz in some brain regions could reflect a harmonic of the true saccadic rhythm rather than direct entrainment.

To directly assess the contribution of harmonic artifacts to our ITC peak distributions, we calculated a harmonic enrichment ratio; the ratio of the observed proportion of ITC peaks at harmonic frequency bins to the proportion expected by chance (27.6% for BBN, 26.7% for LED), with significance determined by bootstrapping (*n* = 1,000 iterations). The results are shown in Supplementary Figure 5. For LED stimulation, harmonic enrichment was significantly below chance for both evoked and modulatory responses (evoked: 0.92×, 95% CI: 0.85–0.98, *p* = 0.016; modulatory: 0.89×, 95% CI: 0.84–0.95, *p* < 0.001), indicating that ITC peaks were preferentially concentrated at non-harmonic frequencies. For BBN stimulation, modest but significant above-chance enrichment was observed for both evoked and modulatory responses (evoked: 1.38×, 95% CI: 1.35–1.41, *p* < 0.001; modulatory: 1.19×, 95% CI: 1.11–1.27, *p* < 0.001). We consider the contrast between BBN and LED results to be informative; if harmonic contamination were a systematic artifact of our analysis pipeline, we would expect similar enrichment patterns for both stimuli. However, we acknowledge that harmonic contamination is not necessarily uniform across all integer multiples of the stimulus frequency and can be concentrated at lower harmonics, and that differences in stimulus properties and auditory vs. visual system filtering characteristics could in principle contribute to the BBN/LED contrast. The BBN and saccade data should therefore be interpreted as consistent with our central conclusions rather than as independent confirmation of them, with the LED data providing the most unambiguous support for the narrowband vs. broadband distinction.

**Figure 5:**
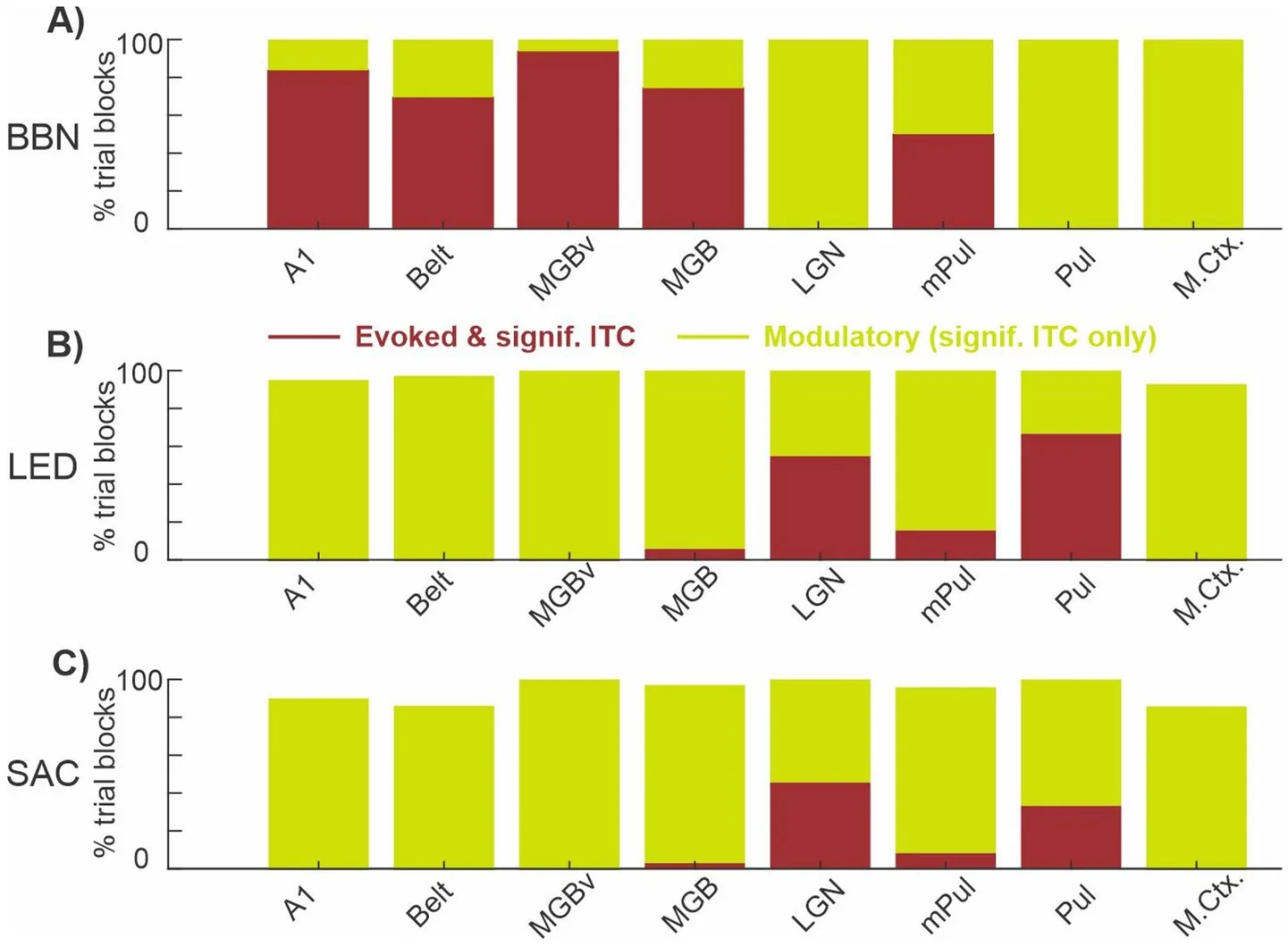
Prevalence of evoked and modulatory responses related to multi-modal events across brain structures. **(A–C)** Bar graphs show the percentage of sites within each brain structure exhibiting significant ITC responses (0–300 ms) for each condition. Brown segments indicate evoked responses (significant ITC accompanied by significant MUA), while green segments indicate modulatory responses (significant ITC without concurrent MUA). **(A)** BBN-related responses. **(B)** LED-related responses. **(C)** Saccade-related responses.

A summary of the results thus far is presented in Figure 5. The height of each bar shows the percentage of sites recorded in each of the eight brain structures that displayed significant ITC in at least one frequency band and at least one linear array electrode contact, while the height of the brown area within the bar illustrates what percentage of those sites also exhibited a concurrent significant MUA response. In other words, the brown areas denote prevalence of evoked type responses (same data as displayed in bar graphs in Figure 2), while the green designates the incidence of modulatory type responses - clustering of significant ITC values at specific frequencies without an accompanying suprathreshold MUA response. In the case of the BBN streams, sites in nearly all areas (with the exception of mPul) exhibited predominantly either evoked or modulatory response profiles (Figure 5A), with the regional distribution summarized in columns 1 and 2 of Table 3. Visual stimulation likewise affected a large proportion of sites in each of the 8 brain structures (Figure 5B), with the corresponding distribution shown in columns 3 and 4 of Table 3. Likewise, during resting-state sites, saccades elicited evoked and modulatory responses across regions (Figure 5C), as summarized in columns 5 and 6 of Table 3. Clearly auditory and visual stimuli, even when they do not match the preferred modality of a specific brain region, absolutely impact neuronal excitability even at a subthreshold level, as evidenced by the total height of each bar approaching 100% in Figures 5A,B. These bar graphs also reveal which of the 8 brain structures show response patterns consistent with membership in the auditory or visual driving pathway, indicated by a predominance of evoked responses (taller brown regions), and which show patterns more consistent with modulatory influences on subthreshold neuronal excitability, indicated by a predominance of modulatory responses (taller green regions). Similarly, inputs related to motor sampling patterns (i.e., saccades) are also capable of impacting neuronal excitability at both sub- and suprathreshold levels as demonstrated by the tallness of the bars in Figure 5C. While it is probable that the modulation seen in visual areas (e.g., LGN and Pul) is due to visual input at saccade end rather than being motor related, modulation in non-visual regions (e.g., A1, MGBv) is less easily explained by visual reafference alone. A previous finding that saccades and visual stimuli reset delta/theta oscillations to opposing excitability phases (O’Connell et al., 2020) suggests that at least in non-visual brain regions saccade-related modulation may reflect a genuine motor contribution. Taken together these observations suggest that saccade-related signals, whether motor, visual reafference, or a combination of both, have the capacity to influence sensory processing across multiple hierarchical levels through the reorganization of ongoing neuronal oscillations.

**Table 3:** Percentage of recording sites exhibiting evoked and modulatory (Modul.) responses across brain regions and stimulus conditions.

Region	BBN		LED		SAC	
	Evoked %	Modul. %	Evoked %	Modul. %	Evoked %	Modul. %
A1	82	18	0	95	0	88
Belt	68	32	0	97	0	84
MGBv	94	6	0	100	0	100
MGB	74	26	6	94	3	94
LGN	0	100	55	45	45	55
mPul	62	38	14	86	10	85

Values represent the proportion of sites within each region classified as evoked or modulatory in response to broadband noise (BBN), LED, and saccade (SAC) conditions.

### Evoked and modulatory responses exhibit distinct AUC patterns across adjacent frequency bands

**Figure 6:**
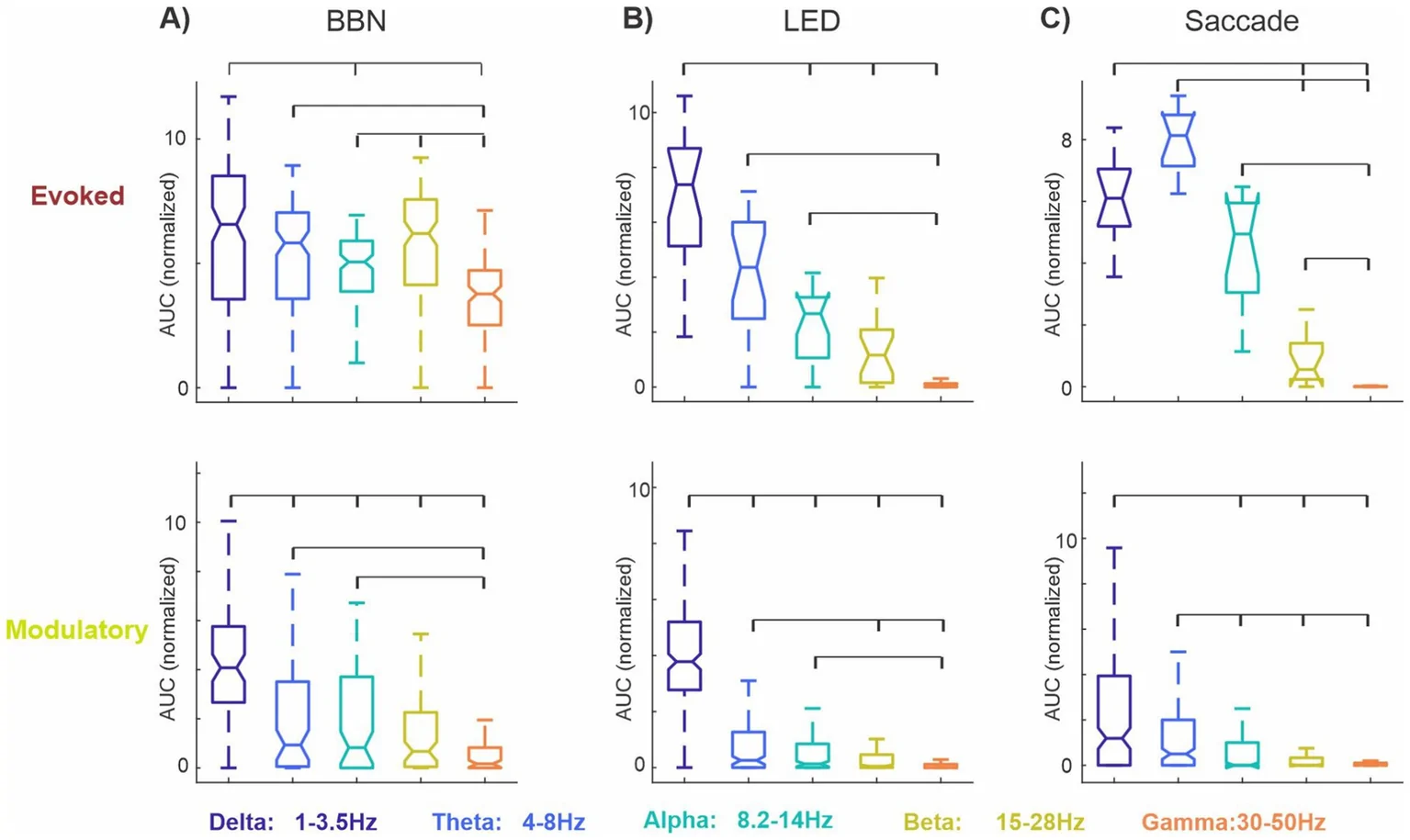
Statistical comparison of frequency band-limited AUCs of significant ITC peak distributions. **(A–C)** Boxplots show pooled area under the curve (AUC) values for the frequency distribution of significant ITC peaks across five canonical frequency bands, separated by response type (evoked, top; modulatory, bottom) and condition. AUCs were computed from sites exhibiting evoked or modulatory responses and pooled across all brain regions. Brackets indicate significant differences between frequency bands (Kruskal–Wallis test with Bonferroni correction, *p* < 0.01). **(A)** BBN-related responses. **(B)** LED-related responses. **(C)** Saccade-related responses.

Given that sites with evoked MUA responses exhibited a qualitatively broader distribution of significant ITC peaks compared to modulatory blocks we sought to quantify how this difference was expressed across neighboring frequency bands. To do so, we computed the area under the curve (AUC) for the frequency distribution of significant ITC peaks within five canonical frequency bands (see colored bars on the x-axes in Figure 4) both response types. Because each site’s ITC peak distribution reflects the frequency at which phase alignment is strongest at that site, the distribution of peak frequencies pooled across sites provides a population-level index of spectral breadth. If phase alignment is broadband, i.e., not constrained to a narrow frequency range, individual sites should peak across a wide range of frequencies, yielding comparable AUCs across neighboring bands. If phase alignment is narrowband, sites should peak within a restricted range, yielding disproportionately large AUCs in one or few bands relative to their neighbors. We used this logic to test whether evoked and modulatory responses differ in spectral breadth. Based on this logic, we hypothesized that evoked responses, reflecting broadband phase alignment, would yield relatively uniform AUCs across adjacent bands. In contrast, we hypothesized that modulatory responses, reflecting narrowband phase alignment, would show disproportionate concentration in specific bands, resulting in significant differences between neighboring frequency bands. To test this, we pooled AUCs within each frequency band by response type (evoked vs. modulatory) across all areas and conducted statistical comparisons across frequency bands. This analysis was performed separately for each event type (Figure 6). For evoked responses to BBN streams, AUC differed significantly across frequency bands overall (Kruskal–Wallis test with Bonferroni correction, *p* < 0.001; Figure 6A, top). Post-hoc comparisons revealed that neighboring low-frequency bands were largely comparable, delta and theta did not differ significantly, with significant differences emerging primarily between non-neighboring bands, driven by gamma differing from lower frequency bands. This pattern is consistent with our hypothesis of relatively broad, uniform phase alignment across low-to-mid frequencies for driving inputs. In contrast, BBN-related modulatory responses showed pronounced differences across adjacent bands, with delta band AUCs significantly exceeding those of all other frequency bands, including the neighboring theta band (*p* < 3.41 × 10^−7^; delta vs. theta: *p* = 3 × 10^−8^; Figure 6A, bottom). Critically, while delta differed significantly from all other bands, theta, alpha, beta and gamma were largely comparable to one another, a delta vs. all-other-bands pattern reflecting the narrowband low-frequency concentration of phase alignment that characterizes modulatory inputs.

For LED-related evoked responses, AUC differed significantly overall (Kruskal-Wallis, *p* < 0.001; Figure 6B, top). Post-hoc comparisons revealed that delta and theta did not differ significantly (*p* = 0.2), with significant differences emerging only between non-neighboring bands (delta vs. alpha, beta and gamma; *p* < 0.001). In contrast, LED-related modulatory responses exhibited strong band-specific effects, with delta band AUCs significantly larger than those of all other frequency bands, including the adjacent theta band (*p* < 1 × 10^−30^; delta vs. theta: *p* = 1 × 10^−31^; Figure 5B, bottom). These statistical comparisons in the context of external sensory stimuli support our hypothesis that evoked responses are characterized by relatively uniform AUCs across neighboring low-frequency bands, whereas modulatory responses show marked band-specific concentrations at delta.

For saccade-related evoked responses, AUC differed significantly overall (Kruskal-Wallis, *p* < 0.001; Figure 6C, top). Although theta band AUCs were the largest, they did not differ significantly from the adjacent delta or alpha bands, with significant differences emerging only between non-neighboring bands (theta vs. beta and gamma; *p* < 1.2 × 10^−34^). This pattern of uniformity across neighboring bands is consistent with that observed for BBN and LED evoked responses. While saccade-related modulatory responses resembled sensory stimulus–related modulatory responses in exhibiting the largest AUCs in the delta band (Figure 6C, bottom), these AUCs were not significantly greater than those in the adjacent theta band (*p* = 0.034). This indicates a broader distribution of ITC peaks across neighboring frequency bands, more similar to the pattern observed for evoked responses than for sensory-driven modulatory responses. This may reflect the aperiodic nature of saccades, variable inter-saccade intervals may produce phase alignment that is broadly distributed across neighboring low-frequency bands rather than concentrated at a single frequency. Alternatively, or additionally, this broader distribution may reflect differences in the underlying circuitry engaged by internally generated motor events compared to externally driven sensory stimuli.

To assess whether the use of different spatial derivations for cortical (CSD) and subcortical (bipolar) recordings influenced these AUC patterns, we repeated the ITC peak detection and subsequent AUC analysis on bipolar-derived cortical signals across all three stimulus conditions (Supplementary Figure 6). The core evoked vs. modulatory AUC distinction was preserved regardless of which spatial derivation was applied, and CSD and bipolar cortical derivations produced highly consistent results across stimulus conditions, confirming that the differing spatial transformations do not confound the spectral profile comparisons presented in Figure 6.

### Spectrotemporal distribution of significant ITC peaks related to multi-modal events across brain structures

**Figure 7:**
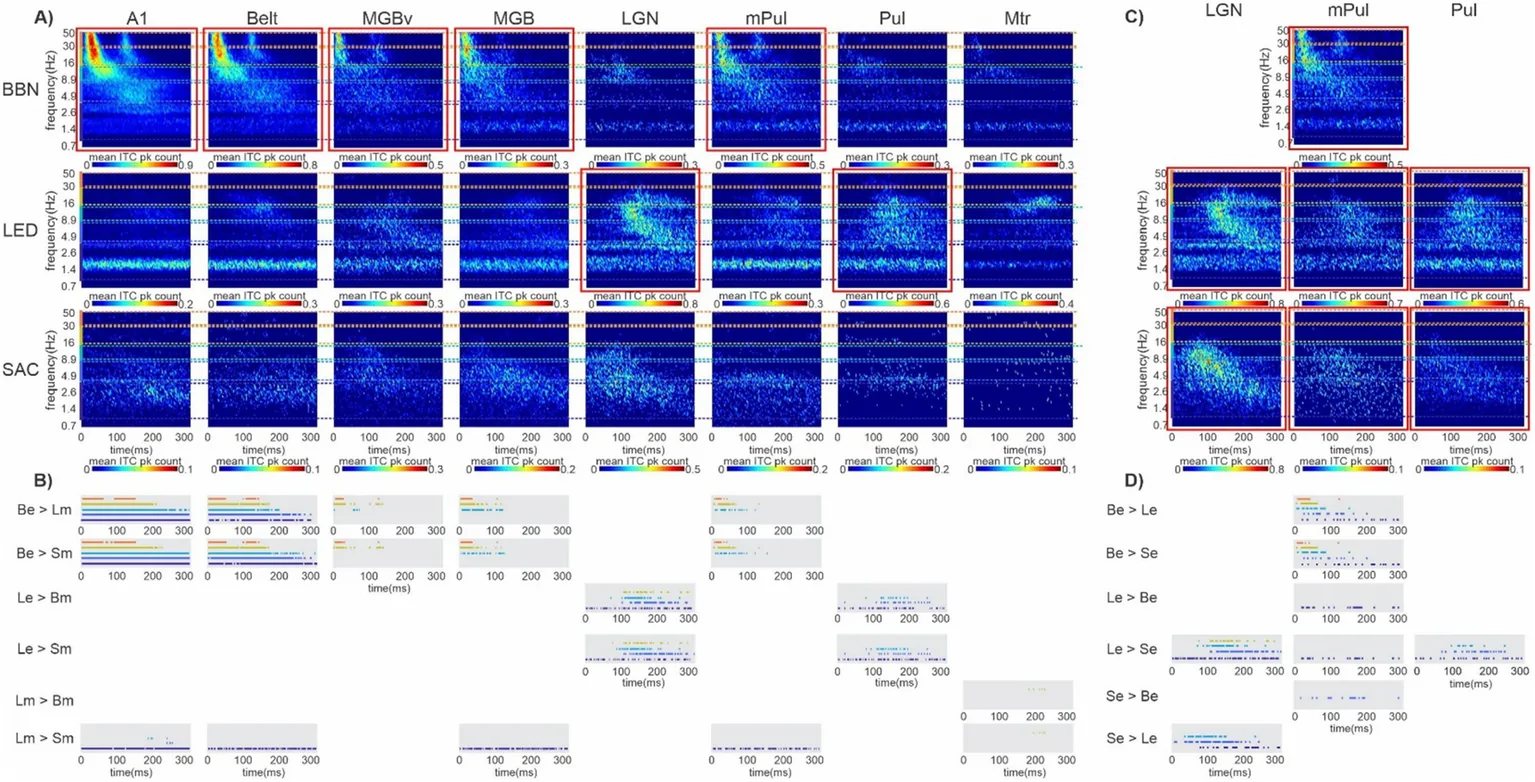
Spectrotemporal distribution of significant CSD and bipolar ITC peaks across brain structures. **(A)** Color plots show the mean spectrotemporal distribution of significant ITC peaks across eight brain regions (columns) for three event types (rows). Red boxes indicate plots derived from sites with evoked MUA responses; unboxed plots reflect modulatory sites. Colored dashed lines on the *y*-axes denote the five frequency bands (see Figures 3, 5). All pairwise comparisons were examined in both directions; non-significant comparisons were omitted for clarity. **(B)** Horizontal lines indicate time intervals where bootstrapped confidence intervals (*p* < 0.05) for ITC peak counts differed significantly between conditions in the direction indicated by the label. Line color denotes frequency band. **(C)** As for **(A)** but showing within-category comparisons for areas where more than one condition produced evoked MUA responses. **(D)** Pairwise comparisons for LGN, mPul, and Pul as in **(B)**. Labels: Be (BBN evoked), Bm (BBN modulatory), Le (LED evoked), Lm (LED modulatory), Se (saccade evoked), Sm (saccade modulatory).

Up to this point, our analysis has focused on the distribution of significant ITC peaks pooled across the entire 0–300 ms post-stimulus period. To gain deeper insight into the temporal dynamics of these responses, we next examined their joint distribution across time and frequency (i.e., their spectrotemporal profile). Figure 7A shows color plots of the mean spectrotemporal distribution of significant ITC peaks within each brain area. In generating this figure, BBN or LED sites that elicited evoked MUA responses were used for regions associated with auditory or visual driving pathways, respectively (i.e., areas with taller brown bars in Figures 5A,B). If no evoked responses were evident, then BBN, LED and saccade modulatory sites were used to construct the color plots. From visual inspection, several features are notable. First, consistent with the Heisenberg uncertainty principle (Cohen, 2020), delta-band ITC peaks are temporally diffuse, spanning the entire 0–300 ms window across all regions. Second, within each area, the timing of ITC peaks varies by event type. For example, in the LGN, LED-related theta/alpha ITC peaks occur ~50 ms later than those associated with saccades, while BBN-related peaks are less frequent and cluster in the alpha band around 100 ms following the stimulus. Third, in BBN sites that elicited evoked MUA responses (highlighted by red boxes), gamma ITC peaks appear in two bursts separated by ~100 ms, likely reflecting transient “on” and “off” MUA responses to the 100 ms BBN stimulus. These gamma bursts are absent in BBN modulatory sites. Finally, saccade-related ITC peaks are predominantly restricted to the delta–theta frequency range across all regions (cf. Figure 4C).

To statistically compare the timing of band limited ITC peaks across the three event types within each area, we calculated simultaneous confidence bands of ITC peak count related to each event type (see Methods) and determined the time intervals in which the bands did not overlap (Figure 7B). All possible pairwise comparisons between conditions were examined and only comparisons showing at least one significant timepoint are shown; non-significant comparisons were omitted for clarity. Within-category comparisons were not always possible due to highly unequal trial counts across evoked and modulatory conditions; in these cases, cross-category comparisons between conditions with more balanced trial counts were used instead. For areas where more than one condition produced evoked responses (LGN, mPul, and Pul), all pairwise comparisons including within-category comparisons are shown in Figures 7C,D.

As expected, in A1 (Figure 7B, first column), BBN sites that elicited evoked responses contained significantly more delta-, theta-, and alpha-band ITC peaks than LED and saccade modulatory sites across the entire 0–300 ms window, i.e., confidence bands did not overlap. In contrast, beta-band ITC peaks were elevated only within the 0–200 ms interval, while gamma-band ITC peaks were elevated within 0–60 ms and 100–150 ms, consistent with transient the “on” and “off” MUA responses to the 100 ms BBN stimulus. LED modulatory sites exhibited significantly more delta-band ITC peaks than saccade modulatory sites across the entire 0–300 ms window, likely reflecting the delta-rate presentation of the LED stream. Theta- and alpha-band LED-related ITC peaks were significantly greater only at later time points, around 250 ms and 200 ms respectively, consistent with delayed visual influences on neural phase coherence in A1. In Belt (Figure 7B, second column), a similar overall pattern was observed.

In the subcortical regions identified as part of the auditory driving pathway (MGBv, MGB, mPul; cf. Figure 5A) greater ITC peak counts in evoked BBN sites compared to LED and saccade modulatory sites were confined to the higher frequency bands (Figure 7B, third, fourth, and sixth columns). In MGBv, beta- and gamma-band ITC peaks were elevated within the 0–40 ms interval and again around 120 ms following stimulus offset. In MGB and mPul, BBN-evoked alpha-band ITC peak counts were also increased relative to LED- and saccade- related modulatory sites during the 0–100 ms duration of the BBN stimulus. In contrast, delta/theta ITC peak counts did not differ between BBN-evoked and LED- or saccade-related modulatory sites, indicating that visual and saccade-related events produce a similar number and temporal distribution of low-frequency ITC peaks as the BBN stimulus. This selective enhancement of higher-frequency ITC peaks in BBN-evoked responses, coupled with comparable low-frequency activity across conditions, suggests a distinction between subcortical and cortical auditory driving pathways.

In the main visual thalamic nucleus, LGN, evoked LED sites contained significantly more delta-band ITC peaks than BBN and saccade modulatory sites across the entire 0–300 ms window (Figure 7B, fifth column), likely reflecting the delta-rate presentation of the LED stream. In addition, theta-, alpha-, and beta-band ITC peaks were elevated during the 100–250 ms, 90–200 ms, and 100–220 ms intervals, respectively, consistent with the delayed onset of LED-evoked responses (cf. Figure 1B).

A similar pattern was observed in Pul (Figure 7B, seventh column), with the exception that beta-band ITC peaks were not increased in LED-evoked sites compared to BBN and saccade modulatory sites. This difference highlights a distinction between LGN and Pul, despite both structures exhibiting robust visual responses and belonging to the visual driving pathway (cf. Figure 5B), suggesting region-specific differences in how visual thalamic nuclei encode stimulus-driven activity across frequency bands.

In Motor cortex, LED modulatory sites contained significantly more beta-band ITC peaks than BBN and saccade modulatory sites within the 190–225 ms (Figure 7B, last column). No significant differences were observed across delta, theta, alpha, or gamma bands. Across all regions, there were no instances in which saccade-related modulatory ITC peak counts exceeded those observed for LED- or BBN-related modulatory sites.

Within-category comparisons are shown in Figures 7C,D. For LGN, the pattern of differences between LED evoked and saccade evoked sites closely mirrored that observed between LED evoked and saccade modulatory sites (Figure 7B, fifth column), though saccade evoked sites showed greater theta- and alpha-band ITC peak counts approximately 50 ms earlier than LED evoked sites, likely reflecting a combination of motor-related preparatory signals preceding the eye movement and visual reafference following it, in contrast to LED-evoked responses which are driven by afferent visual input alone. In mPul, within-evoked comparisons similarly resembled their cross-category counterparts (Figure 7B, sixth column), with the exception of sporadic increases in delta- and theta-band ITC peak counts. In Pul, LED evoked and saccade evoked sites showed a pattern of differences that very closely matched those observed in the corresponding cross-category comparisons (Figure 7B, seventh column).

### Stimulation-rate dependent modulatory phase coherence across frequency bands

**Figure 8:**
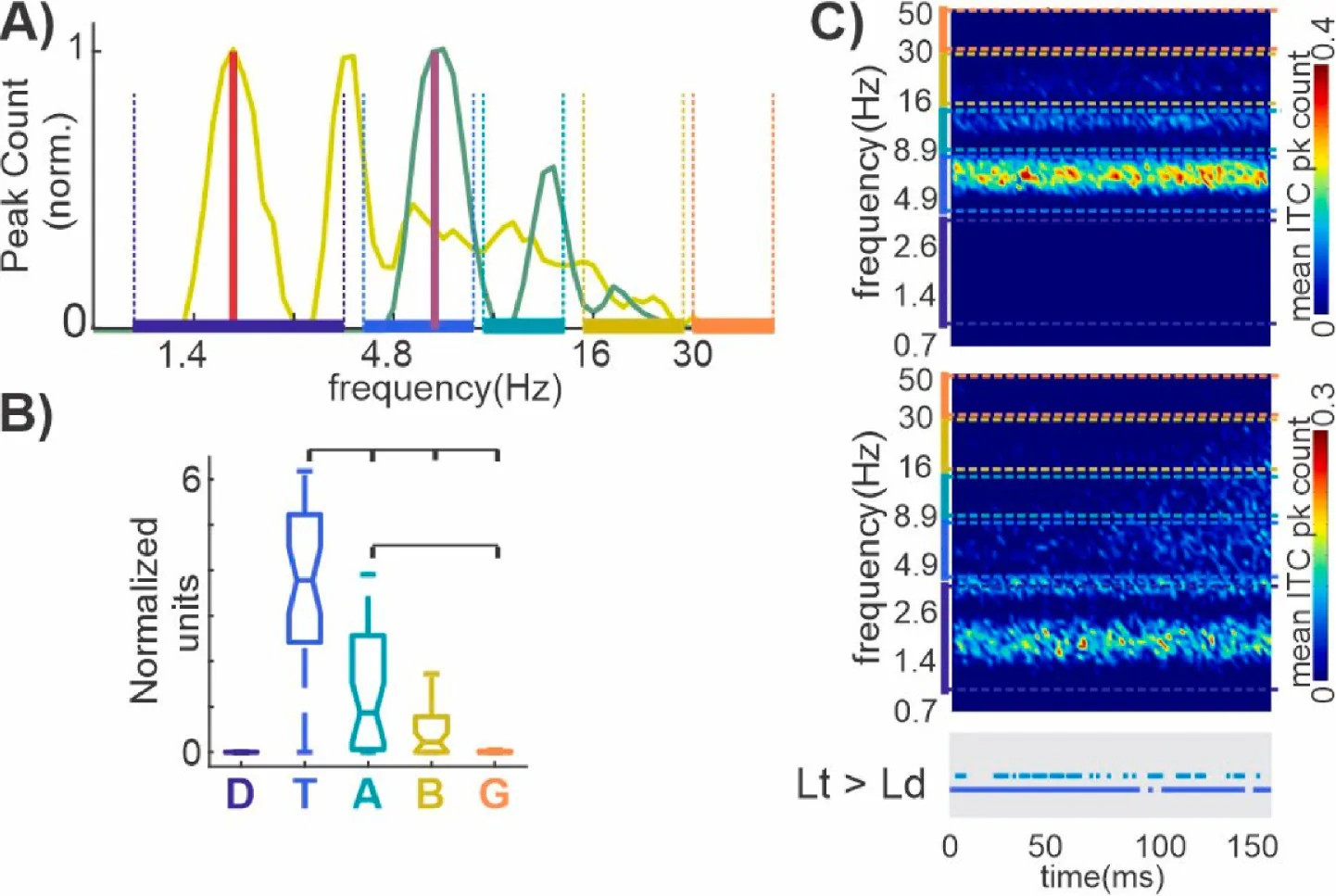
Frequency and spectrotemporal distribution of ITC peaks during theta-rate (6 Hz) LED stimulation. **(A)** Frequency distributions of significant ITC peaks for modulatory LED sites at 6 Hz (dark green) and 1.8 Hz (light green) presentation rates (*n* = 33 sites across six brain regions). Peaks were identified within the 1–50 Hz range and 0–150 ms window. Vertical lines indicate stimulation rates (1.8 Hz, red; 6 Hz, purple). Colored bars along the *x*-axis denote the five frequency bands used in subsequent analyses (see Figures 3, 5). **(B)** Boxplots show pooled AUC values for the frequency distributions of significant ITC peaks from 6 Hz modulatory LED sites, computed across the five frequency bands. Brackets indicate significant differences (Kruskal–Wallis test with Bonferroni correction, *p* < 0.001). **(C)** Spectrotemporal distributions of significant ITC peaks for modulatory LED sites at 6 Hz (top) and 1.8 Hz (bottom). Colored bars on the *y*-axes indicate frequency bands. Within the gray box, horizontal lines mark time intervals where bootstrapped confidence intervals (*p* < 0.05) for theta- and alpha-band ITC peaks differ between 6 Hz (Lt) and 1.8 Hz (Ld) conditions.

The present findings demonstrate that, for modulatory responses, the largest proportion of significant ITC peaks is concentrated in the delta frequency band for BBN and LED stimuli, and in the delta–theta range for saccades (cf. Figures 4, 7), consistent with neural oscillatory entrainment. To more directly test whether modulatory entrainment can be driven beyond the delta range, rhythmic streams of LED flashes were presented at a theta stimulation rate of 6 Hz in a subset of recordings (*n* = 33; 5 A1, 4 Belt, 4 MGBv, 7 MGB, 7 mPul, and 6 Motor cortex sites). Traces in Figure 8A show the frequency distribution of significant ITC peaks related to the 6 Hz LED stream (dark green trace), overlaid with the corresponding distribution obtained from the same sites during the 1.8 Hz LED stream (light green trace). For visualization purposes, both distributions were normalized to the peak of each trace. Similar to the 1.8 Hz condition, the distribution associated with the 6 Hz stream exhibits a pronounced cluster of significant ITC peaks at the stimulation rate (purple solid line), consistent with entrainment. A secondary concentration of peaks is evident near 12 Hz, likely reflecting a harmonic relationship, with additional grouping in the beta range around 18 Hz. As with the 1.8 Hz LED stream, few ITC peaks were observed in the gamma band. Notably, the 6 Hz condition revealed no ITC peaks in the delta band, indicating that phase alignment of oscillatory frequencies lower than the stimulation rate does not appear to occur.

Next, we examined whether the frequency signatures of modulatory phase alignment elicited under theta rate stimulation differed from that observed during modulatory responses to the delta rate LED stream within these six brain structures (cf. Figure 6). As shown in Figure 8B, theta-band AUCs associated with the 6 Hz LED related modulatory responses were significantly greater than those of all other frequency bands, including the adjacent alpha band (*p* < 0.001, Kruskal–Wallis test with Bonferroni correction). These results indicate that modulatory responses, irrespective of the stimulation rate by which they are generated, exhibit a preferential concentration of ITC peaks within a specific frequency band.

Color plots in Figure 8C show the mean spectrotemporal distribution of significant ITC peaks constructed form the same modulatory 6 Hz (top) and 1.8 Hz (bottom) LED sites. Note that due to the rapid presentation of the 6 Hz LED flashes (SOA = 166 ms) the timescale of these plots is half that of those in Figure 7. Again, it is apparent that there were no ITC peaks observed in the delta range during theta-rate LED stimulation. As expected, theta-rate blocks exhibited significantly more theta-band ITC peaks than delta-rate blocks across the entire 0-150 ms window (blue horizontal line within grey box), reflecting the rhythmic theta presentation rate of the LED stream. In addition, alpha-band ITC peaks were significantly greater, especially around 20-70 ms post LED flash (green horizontal line within grey box), indicating that theta-rate LED stimulation induces earlier phase coherence in both theta- and alpha-frequency bands compared to delta-rate stimulation.

### Phase alignment of MUA across brain structures related to multi-modal events

**Figure 9:**
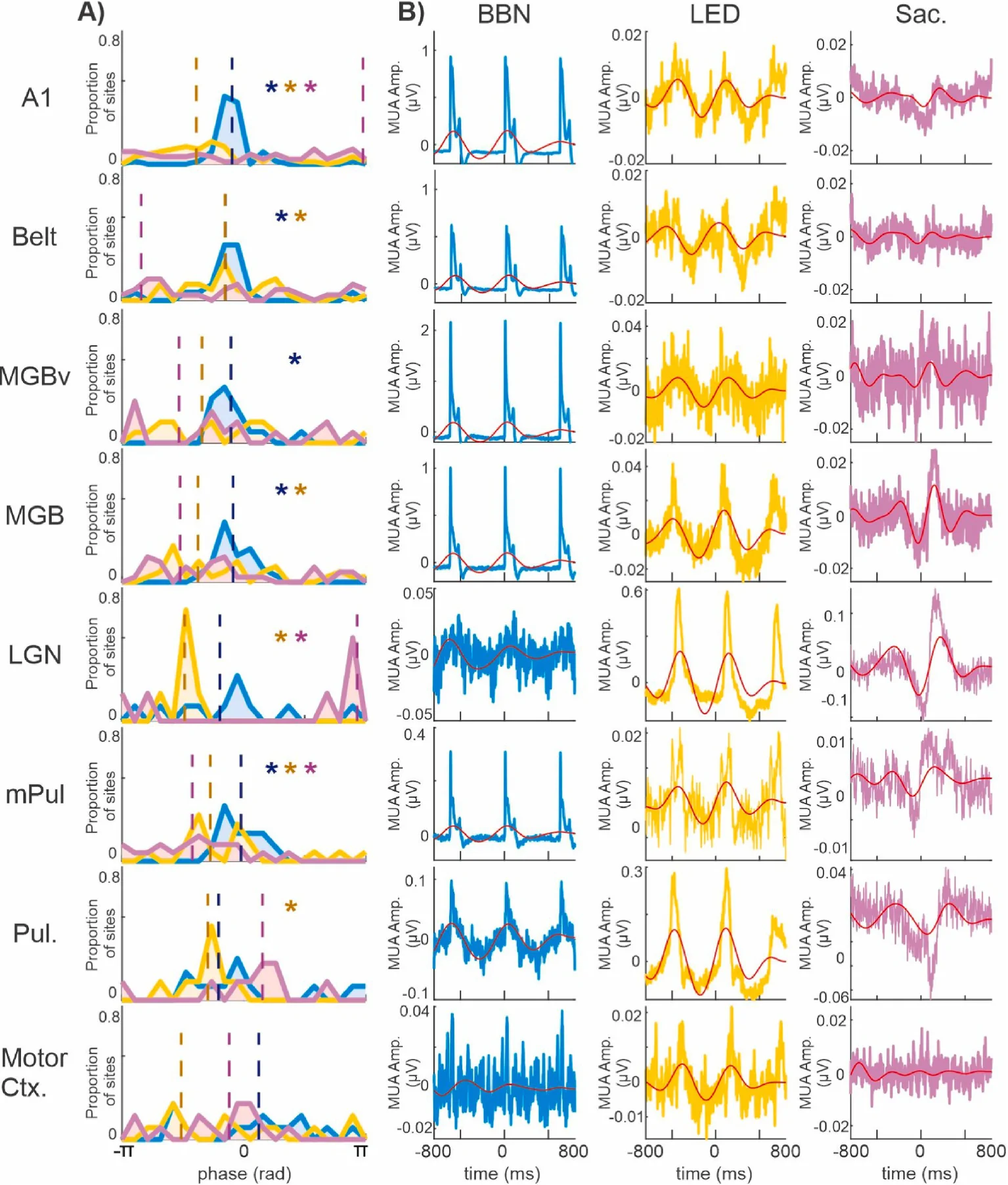
Phase organization of low-frequency MUA activity at the onset of multimodal events across brain structures **(A)** At event onset for BBN bursts (blue), LED flashes (yellow), and saccades (pink), pooled across recording sites within each structure. Phases were computed at the frequency of peak ITC within the delta band for BBN and LED, and within the delta theta range for saccades. Vertical lines indicate circular mean phase (color coded by event type). Colored asterisks denote significant phase bias (Rayleigh’s test, *p* < 0.05). **(B)** Event-aligned MUA traces for BBN (blue), LED (yellow), and saccades (pink), averaged across sites within each structure. Red traces show bandpass-filtered MUA (1.2–2.4 Hz for BBN and LED; 1–5 Hz for saccades).

Results so far show that neuronal oscillations entrain with a similar excitability phase, as indexed by significant ITC values, to the rate of the rhythmically presented external stimuli and quasi-rhythmic saccades. Our next question centered around whether oscillations were being synchronized to a similar high or low excitability phase across areas. Such synchronization could be behaviorally relevant as a means to perhaps facilitate or impede the transfer of sensory information. To investigate this, since MUA has a straightforward relationship with excitability (Lakatos et al., 2013; O’Connell et al., 2020, 2011), we calculated the mean MUA phase at the frequency of peak ITC values. This was done within the delta band for the BBN and LED stimulus streams, and within the delta-theta band for saccade-related activity. Figure 9A shows stacked colored histograms depicting the distribution of mean translaminar and transnuclei MUA phases for each recording site at event onset. In generating this figure, BBN or LED trial blocks that elicited evoked MUA responses were used for brain structures considered part of the auditory or visual driving pathways (i.e., areas with taller brown bars in Figures 5A,B) respectively. Otherwise, BBN, LED and saccade modulatory trial blocks were used to construct the phase histograms. Colored asterisks indicate brain areas exhibiting significant phase bias, as determined by Rayleigh’s test for non-uniformity (*p* < 0.01).

During BBN stimulation, we observed a significant non-random distribution of mean phases in five of the eight recorded brain areas (A1: *p* = 3 × 10^−17^; Belt: *p* = 1 × 10^−6^; MGBv: *p* = 1 × 10^−6^; MGB: *p* = 1 × 10^−9^; and mPul: *p* = 1 × 10^−6^). In these regions, which notably are main nodes of the auditory driving pathway, the mean phase corresponded to a high-excitability state, as indicated by the grand average translaminar/transnuclei MUA reaching its positive peak - reflecting elevated neuronal firing - at the time of the BBN bursts (Figure 9C, blue traces). Moreover, the mean MUA phase distributions across these five areas did not differ significantly (Fisher’s test for equality of circular means, *p* > 0.05), suggesting that excitability was aligned across the auditory thalamocortical network. Such phase alignment likely maximizes the probability of successful signal transmission between auditory-related thalamic and cortical sites by ensuring that interconnected regions reach peak excitability within the same temporal window, thereby facilitating efficient information flow. In contrast, LGN, Pul, and motor cortex, which are part of the auditory modulatory pathway, exhibited a random distribution of mean phases, suggesting that BBN related delta oscillations in these areas did not consistently align to a common excitability phase across trial blocks.

During LED stimulation, pooled mean delta-band MUA phases were significantly clustered in A1 (*p* = 3 × 10^−6^), Belt (*p* = 2 × 10^−4^), MGB (*p* = 0.01) and mPul (*p* = 0.01), regions associated with the visual modulatory pathway. Like the pattern observed during BBN stimulation, this clustering occurred around the high-excitability phase, as indicated by the grand average translaminar/transnuclei MUA from these five areas approaching its positive peak at the time of the LED flashes (Figure 9C, yellow traces). Consistent with coordinated entrainment to the LED stream across these areas, the mean MUA phase distributions did not differ significantly (Fisher’s test for equality of circular means, *p* > 0.05). Regarding the visual driving pathway, MUA signals in the LGN (*p* = 5 × 10^−4^) and Pul (*p* = 1 × 10^−4^) also exhibited significant phase clustering (Rayleigh’s uniformity test), and were not significantly different neither from each other or the four areas listed above in the visual modulatory pathway (Fisher’s test for equality of circular means, *p* > 0.05). Mean delta MUA phases from recording sites in MGBv and motor cortex did not show a non-random phase distribution (Rayleigh’s uniformity tests, *p* > 0.05).

During resting-state saccade-related activity, pooled mean delta/theta MUA phases were significantly clustered in A1 (*p* = 0.02), LGN (*p* = 0.002) and mPul (*p* = 0.004). The angular means of A1 and LGN were nearly identical (3.07 and 2.92 rad, respectively; Fisher’s test for equality of circular means, *p* = 0.7), whereas the mean phase in mPul was significantly different (−1.33 rad; Fisher’s test, *p* < 0.0001). These results indicate that saccades entrain delta/theta oscillations in A1 and LGN to a low-excitability phase, as reflected by the grand average translaminar/transnuclear MUA reaching its negative peak at saccade onset (Figure 9C, pink traces). In contrast, delta/theta oscillations in mPul are entrained to a slightly more excitable phase, with MUA just beginning to approach its positive peak, suggesting a phase offset relative to A1 and LGN. Mean delta/theta MUA phases from the remaining five regions did not exhibit significant clustering (Rayleigh’s tests, *p* > 0.05).

## Discussion

### Functional differentiation of driving and modulatory circuits

The present study provides direct physiological evidence for a functional distinction between driving and modulatory circuits across cortical and subcortical sensory structures in the non-human primate brain. By jointly quantifying suprathreshold (evoked MUA) and subthreshold (narrowband ITC) activity, we demonstrate that auditory, visual, and motor sampling events can elicit two separable classes of neural responses within an area. Preferred sensory stimuli produce broadband phase alignment, reflected in the uniform area under the curves (AUCs) across adjacent frequency bands (Figure 6), and are accompanied by significantly increased multiunit firing (Figure 2). Together, these features are consistent with driving inputs that convey sensory content through strong suprathreshold activation. In contrast, non-preferred sensory and internally generated motor events produced narrowband, frequency-specific ITC modulation - typified by a dominant AUC peak at the rate of stimulation or event occurrence (Figures 6, 8) - without corresponding increases in firing (Figure 5). This pattern is consistent with modulatory inputs that regulate ensemble excitability rather than transmit sensory content. These distinctions are consistent with the conceptual framework of Sherman and Guillery (1998) and Sherman and Guillery (2002), who proposed that driving pathways transmit the main information content while modulatory pathways control their efficacy. Our results provide candidate spectrotemporal signatures that may reflect these circuit properties across cortical and thalamic levels in primates, though future studies combining passive and active paradigms will be needed to directly establish their relevance for behavior and perception. Furthermore, by examining these eight brain structures we identify which regions show response patterns consistent with driving, cross-modal modulatory, or saccade related modulatory circuit properties (Figure 5). Additionally, our results reveal that multi-modal events induce coordinated phase alignment of neuronal excitability across cortical and subcortical structures, indicating that rhythmic sensory and saccade-related signals dynamically synchronize network excitability (Figure 9), which may facilitate efficient cross-modal information flow (e.g., Lakatos et al., 2007).

While the spectral signatures described here were established in cases where the driving vs. modulatory distinction is well-constrained by anatomy and prior literature, they may provide a useful framework for more general classification of driving and modulatory signals in future work. A natural first step would be to examine how selective attention modulates these signatures in the areas studied here, would itself be theoretically informative, and would generate a richer set of reference signatures encompassing both passive and attentional states. Beyond this, cross-species validation in rodents, where genetic tools allow direct manipulation of specific thalamocortical circuit elements, could establish whether the signatures generalize across species and could provide causal evidence linking specific circuit mechanisms to the broadband and narrowband spectral profiles we describe. Finally, the signatures may have clinical translational value: intracranial recordings in human epilepsy patients offer an opportunity to test whether the same spectral fingerprints can classify driving and modulatory inputs in the human brain, potentially providing a non-invasive tool for characterizing circuit dysfunction in conditions where thalamocortical communication is disrupted. Figure 6 provides a quantitative benchmarking template that could support such comparisons in future work.

### Subthreshold modulation as a widespread neural mechanism

A striking feature of the data is the apparent ubiquity of subthreshold modulation across structures and event types. Even in regions not classically associated with the eliciting modality, consistent phase-locked changes in oscillatory activity were observed (see Figures 5, 7). This suggests that cross-modal and saccade-related signals exert widespread modulatory control, dynamically shaping the excitability of sensory networks. Extensive work in humans and non-human primates has established cross-modal modulation of sensory cortices (reviewed in Choi et al., 2023; Ghazanfar and Schroeder, 2006; Kayser and Logothetis, 2007; Lakatos et al., 2009; Mégevand et al., 2020) and motor cortex (Chen et al., 2008; Popescu et al., 2004). Previous primate studies have reported suprathreshold audiovisual integration in the pulvinar (Vittek et al., 2025; Vittek et al., 2023), auditory, visual, and somatosensory responses in the superior colliculus (Wallace and Stein, 1994) and human imaging work has identified enhanced LGN and MGB responses during audiovisual co-stimulation (Noesselt et al., 2010). More recently, visual modulation of auditory-related activity was observed in the macaque inferior colliculus (Schmehl et al., 2025). Subthreshold modulation via phase alignment of the local field potential has been posited as a mechanism to mediate such multisensory effects but has generally been studied in the cortex (e.g., Lakatos et al., 2007, 2008, 2009). Our findings extend the subcortical multisensory literature by demonstrating that oscillatory phase alignment is a prominent feature of cross-modal processing in subcortical nuclei.

It is well documented in primates that self-generated motor behaviors - such as eye movements, locomotion, and vocalization - modulate neural activity across multiple brain regions, including visual cortex (reviewed in Gegenfurtner, 2016), auditory cortex (Eliades and Wang, 2003; Leszczynski et al., 2023; O’Connell et al., 2020), somatosensory cortex (Confais et al., 2017; Leszczynski et al., 2023), motor cortex (Cheung et al., 2016), LGN (Ramcharan et al., 2001; Sylvester et al., 2005), pulvinar nuclei (Robinson et al., 1990; L. Schneider et al., 2023) and inferior colliculus (Groh et al., 2001). Consistent with our findings, these studies generally report a suppression of population-level activity associated with self-produced actions (Brooks and Cullen, 2019; Lohse et al., 2022). Our study complements these prior reports by demonstrating that motor-related modulation of sensory activity arises through an alignment of the excitability phase of low-frequency oscillations. Furthermore, to our knowledge, this is the first evidence that eye movements modulate neuronal activity within the MGB nuclei in primates.

These modulatory effects were most prominent in low-frequency bands, consistent with entrainment to the rates of stimulus presentation (1.6, 1.8, and 6 Hz) and to the saccadic rhythm (~3 Hz). While a previous study in primate auditory cortex demonstrated entrainment to preferred modality stimuli outside the delta range (Lakatos et al., 2013), our findings extend this work by showing that non-preferred modality stimuli can also entrain neural activity beyond the delta range (i.e., in the theta band). Additionally, we found no significant ITC peaks at frequencies lower than the stimulation rate, indicating that phase alignment does not occur at subharmonics of the stimulation frequency.

### Spectral signatures of driving and modulatory influences

The spectral characteristics of evoked and modulatory responses further clarify their mechanistic distinctions. Consistent with the framework described above, evoked responses exhibited broadband ITC across adjacent frequency bands (Min et al., 2007; Palva et al., 2005; Shah et al., 2004). This broadband phase alignment likely arises from the strong, convergent synaptic input characteristic of driving pathways, though we note that broadband low-frequency increases can reflect both synchronous activation of diverse neuronal populations and non-synchronized firing-rate modulations (Vinck et al., 2023). In contrast, previous studies in humans and non-human primates have shown that cross-modal responses that do not elicit suprathreshold activity, instead, elicit modulatory-type responses, which are characterized by frequency-specific phase locking rather than broadband synchronization (Lakatos et al., 2007; Kayser et al., 2008; Mercier et al., 2013; Thorne et al., 2011). Our findings confirm and extend these reports by demonstrating that modulatory responses in both sensory cortices and subcortical structures, produced by non-preferred sensory and saccade-related events, exhibit phase locking that is tightly aligned with the rate of stimulation for externally driven events (BBN and LED), but extends across neighboring low-frequency bands for internally generated events such as saccades. This pattern suggests that externally driven inputs impose precise temporal structure, whereas internally generated signals engage broader low-frequency coordination. However, we acknowledge that the external vs. internal distinction is confounded with signal bandwidth in the current dataset, unlike the fixed-frequency periodic stimulation of BBN and LED, the variable inter-saccade intervals and aperiodic nature of saccades may result in phase alignment that is less concentrated at a single frequency and more broadly distributed across neighboring low-frequency bands.

This oscillatory entrainment appears to be mediated by modulatory inputs that shape membrane potential fluctuations without engaging extensive neuronal firing. Such narrowband synchronization may arise through feedback projections from higher-order cortical or thalamic regions, consistent with the known physiology of matrix-type thalamocortical cells and cortico-thalamic modulatory loops (Jones, 2001; Sherman and Guillery, 2011). This aligns with the view that neural communication depends on rhythmic coordination rather than sustained firing (Engel et al., 2001; Fries, 2005). Within this framework, driving and modulatory circuits act synergistically: driving inputs convey the content of communication, while modulatory inputs regulate its temporal precision.

### Relationship to the spectrolaminar framework

A related but distinct framework for characterizing cortical circuit function is the spectrolaminar framework proposed by Bastos et al. (2015) and Mendoza-Halliday et al. (2024), which links feedforward and feedback processing to distinct laminar oscillatory signatures: gamma-band activity in superficial layers is proposed to index feedforward processing, while beta-band activity in deep layers is proposed to index feedback processing. Our evoked/modulatory framework and the spectrolaminar framework may be best understood as complementary rather than duplicative, as they operate at different levels of analysis and capture related but distinct aspects of circuit communication. Conceptually, the alignment between the two is intuitive: feedforward and driving inputs have historically been considered synonymous, and in that sense the two frameworks are theoretically consistent. However, the specific spectral signatures identified by each do not directly overlap. Bastos et al. (2015) and Mendoza-Halliday et al. (2024) identify beta oscillations in deep layers as the signature of feedback processing, whereas our modulatory signature is characterized by narrowband, delta-dominated ITC aligned to stimulus and saccade rates. This discrepancy may reflect differences in methodology and in the aspects of modulatory communication each framework is sensitive to; in particular, those studies used raw LFP, whereas we used CSD and bipolar derivations, which offer greater spatial precision and reduced susceptibility to volume conduction.

Because we did not perform layer-specific analyses, we cannot directly speak to the laminar profile of our identified signatures, though prior work in primary auditory cortex indicates that modulatory responses are heavily biased toward superficial layers (Lakatos et al., 2007; O’Connell et al., 2020). Our framework also extends beyond the cortical circuits addressed by the spectrolaminar literature to subcortical structures such as the MGB, LGN, and pulvinar, where organization differs fundamentally from cortex and where the feedforward/feedback framework has not been systematically applied. Directly comparing the two frameworks, assessing where they converge and where they diverge, represents a promising direction for future work.

### Limitations of cross-condition comparisons

A limitation of the current study is that the auditory and visual stimuli were not perfectly matched across several dimensions, including pulse duration (100 ms for BBN vs. 25 ms for LED), intensity, and spatial precision of retinal stimulation. Eye position was tracked but not formally controlled during visual stimulation, meaning the position of the LED relative to the fovea may have varied across trials and subjects. Differences in stimulus salience arising from these factors could in principle have contributed to some of the differences observed between BBN and LED conditions, as more salient stimuli are known to capture attention more effectively, which can itself influence neural phase alignment (Lakatos et al., 2009). We note however that the core analyses were conducted separately for each stimulus condition and that direct quantitative comparisons between BBN and LED response magnitudes were not a primary focus of the study.

### Harmonic artifact considerations

A potential concern with any ITC analysis using periodic stimulation is the contribution of harmonic artifacts, whereby non-sinusoidal neural responses to rhythmic stimulation produce power at integer multiples of the stimulus frequency that may be misinterpreted as genuine oscillatory activity (Cole and Voytek, 2019; Giehl et al., 2021). Our harmonic enrichment analysis provides the clearest evidence against harmonic contamination for the LED data, where both evoked and modulatory responses showed harmonic enrichment significantly below chance, indicating that ITC peaks were preferentially concentrated at non-harmonic frequencies (Supplementary Figure 5). This provides unambiguous support for our central narrowband vs. broadband distinction in the visual domain.

For BBN stimulation, the picture is more nuanced. Modest above-chance harmonic enrichment was observed for both evoked and modulatory responses, and we acknowledge that harmonic contamination concentrated at lower harmonics could in principle contribute to the delta-dominated ITC pattern observed for BBN modulatory responses. Differences in stimulus properties and the distinct low-pass filtering characteristics of the auditory and visual systems may also contribute to the contrasting BBN and LED enrichment patterns. We therefore treat the BBN and saccade data as consistent with our central conclusions rather than as independent confirmation of them, with the LED data providing the most unambiguous support for the narrowband vs. broadband distinction.

### Motor - sensory modulation and predictive timing

Internally generated saccades produced clear modulatory signatures: delta/theta ITC peaks without associated suprathreshold responses. Previous studies in primates have shown that eye movements result in changes in oscillatory activity in sensory cortical areas and hippocampus (Benedetto et al., 2020; Hoffman et al., 2013; Jutras et al., 2013; Leszczynski et al., 2025; O’Connell et al., 2020). We add to the literature by showing that eye movements also modulate oscillatory activity in MGB and Pulvinar nuclei. These results suggest that motor-related inputs act primarily through modulatory mechanisms that pre-tune sensory circuits for impending changes in input. The phase alignment induced by saccades may serve to align cortical and thalamic excitability states with expected sensory consequences of movement - a form of predictive timing that minimizes sensory interference during self-generated actions (Flinker et al., 2010; O’Connell et al., 2020; Schneider et al., 2014). Additionally, the rebound in excitability following motor inputs is positioned to indirectly enhance sensory processing (Barczak et al., 2019). This interpretation resonates with models of active sensing, in which motor acts synchronize sensory sampling with behaviorally relevant environmental rhythms (Helfrich, 2018; Melloni et al., 2009; Schroeder et al., 2010).

Interestingly, phase alignment across LGN, A1 and mPul following saccades was not perfectly synchronous, with mPul exhibiting a more excitable phase at saccade onset. This pattern suggests that distinct network nodes may experience systematic phase lags consistent with hierarchical processing delays. Supporting this idea, a previous macaque study showed that microstimulation of mPul prior to visual target onset alters saccadic reaction times (Dominguez-Vargas et al., 2017), indicating a causal role in shaping saccade timing and visuomotor integration rather than merely reactive processing. Such controlled asynchrony could allow the system to sequentially gate information flow, ensuring that sensory processing unfolds in a temporally ordered manner following each motor event.

### Effect of spatial derivation on ITC signatures

The use of different spatial derivations for cortical (CSD) and subcortical (bipolar) recordings warrants consideration, as these transformations have different spatial filtering properties, CSD acts as a stronger high-pass spatial filter than bipolar derivation and more effectively attenuates spatially broad volume-conducted signals (Nicholson and Freeman, 1975; Mitzdorf, 1985; Kajikawa and Schroeder, 2011). In principle this could narrow cortical spectral profiles relative to subcortical profiles. However, as demonstrated in Supplementary Figure 6, the core evoked vs. modulatory distinction was preserved regardless of which spatial derivation was applied to cortical sites, and CSD and bipolar cortical derivations produced highly consistent results across stimulus conditions. We therefore conclude that the differing spatial transformations do not systematically confound our comparison of ITC signatures between cortical and subcortical structures.

## Conclusion

In summary, our findings delineate the physiological organization of driving and modulatory circuits across cortical and subcortical sensory hierarchies. Our results support an integrative model in which driving and modulatory circuits cooperate through oscillatory phase dynamics to regulate sensory processing. Driving inputs convey content via synchronous firing, while modulatory circuits define the temporal windows during which such transmission is effective. Through phase alignment, modulatory pathways can transiently couple regions into coherent processing assemblies, while phase divergence may limit or gate information transfer. This dynamic interplay allows the brain to flexibly route sensory information according to behavioral demands and contextual predictions. By combining measures of suprathreshold and subthreshold activity, our results extend classical anatomical distinctions and link these circuit types to distinct electrophysiological signatures that can be tracked across cortical and thalamic hierarchies. Finally, extending these analyses to behaviorally engaged states could determine how attention or expectation modulate the balance between driving and modulatory activity. Such experiments would provide crucial tests of whether phase coordination serves as an active mechanism for sensory gating and predictive control within the context of driving and modulatory circuit interactions.

## Data Availability

The raw data supporting the conclusions of this article will be made available by the authors, without undue reservation.

## References

[ref1] Barczak A. Haegens S. Ross D. A. McGinnis T. Lakatos P. Schroeder C. E. (2019). Dynamic modulation of cortical excitability during visual active sensing. Cell Rep. 27, 3447–3459.e3. doi: 10.1016/j.celrep.2019.05.072, 31216467 PMC6598687

[ref2] Barczak A. O’Connell M. N. McGinnis T. Ross D. Mowery T. Falchier A. . (2018). Top-down, contextual entrainment of neuronal oscillations in the auditory thalamocortical circuit. Proc. Natl. Acad. Sci. USA 115, E7605–E7614. doi: 10.1073/pnas.1714684115, 30037997 PMC6094129

[ref3] Barczak A. O’Connell M. N. Schroeder C. E. (2023). “Thalamic contributions to multisensory convergence and processing,” in The Cerebral Cortex and Thalamus, eds. Usrey W. M. Sherman S. M. (New York: Oxford University Press), 305–316.

[ref4] Bastos A. M. Vezoli J. Bosman C. A. Schoffelen J.-M. Oostenveld R. Dowdall J. R. . (2015). Visual areas exert feedforward and feedback influences through distinct frequency channels. Neuron 85, 390–401. doi: 10.1016/j.neuron.2014.12.018, 25556836

[ref5] Benedetto A. Morrone M. C. Tomassini A. (2020). The common rhythm of action and perception. J. Cogn. Neurosci. 32, 187–200. doi: 10.1162/jocn_a_01436, 31210564 PMC6938410

[ref6] Berg D. J. Boehnke S. E. Marino R. A. Munoz D. P. Itti L. (2009). Free viewing of dynamic stimuli by humans and monkeys. J. Vis. 9, 1–15. doi: 10.1167/9.5.19, 19757897

[ref7] Besle J. Schevon C. A. Mehta A. D. Lakatos P. Goodman R. R. McKhann G. M. . (2011). Tuning of the human neocortex to the temporal dynamics of attended events. J. Neurosci. 31, 3176–3185. doi: 10.1523/JNEUROSCI.4518-10.2011, 21368029 PMC3081726

[ref8] Biagioni T. Fratello M. Garnier E. Lagarde S. Carron R. Medina Villalon S. . (2025). Interictal waking and sleep electrophysiological properties of the thalamus in focal epilepsies. Brain Commun. 7:fcaf102. doi: 10.1093/braincomms/fcaf102 40135069 PMC11934066

[ref9] Brooks J. X. Cullen K. E. (2019). Predictive sensing: the role of motor signals in sensory processing. Biol. Psychiatry: Cognit. Neurosci. Neuroimaging 4, 842–850. doi: 10.1016/j.bpsc.2019.06.003, 31401034 PMC6733654

[ref10] Brosch M. Selezneva E. Scheich H. (2005). Nonauditory events of a behavioral procedure activate auditory cortex of highly trained monkeys. J. Neurosci. 25, 6797–6806. doi: 10.1523/JNEUROSCI.1571-05.2005, 16033889 PMC6725347

[ref11] Cappe C. Rouiller E. M. Barone P. (2009). Multisensory anatomical pathways. Hear. Res. 258, 28–36. doi: 10.1016/j.heares.2009.04.017, 19410641

[ref12] Chen J. L. Penhune V. B. Zatorre R. J. (2008). Listening to musical rhythms recruits motor regions of the brain. Cereb. Cortex 18, 2844–2854. doi: 10.1093/cercor/bhn042, 18388350

[ref13] Cheung C. Hamiton L. S. Johnson K. Chang E. F. (2016). The auditory representation of speech sounds in human motor cortex. eLife 5:e12577. doi: 10.7554/eLife.12577 26943778 PMC4786411

[ref14] Choi I. Demir I. Oh S. Lee S.-H. (2023). Multisensory integration in the mammalian brain: diversity and flexibility in health and disease. Philos. Trans. R. Soc. Lond. Ser. B Biol. Sci. 378:20220338. doi: 10.1098/rstb.2022.0338, 37545309 PMC10404930

[ref15] Cohen L. (2020). “Time-frequency analysis: what we know and what we Don’t,” in Landscapes of Time-Frequency Analysis: ATFA 2019, eds. Boggiatto P. Bruno T. Cordero E. Feichtinger H. G. Nicola F. Oliaro A. (Cham: Springer International Publishing), 75–101.

[ref16] Cole S. Voytek B. (2019). Cycle-by-cycle analysis of neural oscillations. J. Neurophysiol. 122, 849–861. doi: 10.1152/jn.00273.2019, 31268801

[ref17] Confais J. Kim G. Tomatsu S. Takei T. Seki K. (2017). Nerve-specific input modulation to spinal neurons during a motor task in the monkey. J. Neurosci. 37, 2612–2626. doi: 10.1523/JNEUROSCI.2561-16.2017, 28159911 PMC6596638

[ref18] Dominguez-Vargas A.-U. Schneider L. Wilke M. Kagan I. (2017). Electrical microstimulation of the Pulvinar biases saccade choices and reaction times in a time-dependent manner. J. Neurosci. 37, 2234–2257. doi: 10.1523/JNEUROSCI.1984-16.2016, 28119401 PMC5338763

[ref19] Eliades S. J. Wang X. (2003). Sensory-motor interaction in the primate auditory cortex during self-initiated vocalizations. J. Neurophysiol. 89, 2194–2207. doi: 10.1152/jn.00627.2002, 12612021

[ref9001] Engbert R. Kliegl R. (2003). Microsaccades uncover the orientation of covert attention. Vision Research. 43, 1035–1045. doi: 10.1016/s0042-6989(03)00084-1, 12676246

[ref20] Engel A. K. Fries P. Singer W. (2001). Dynamic predictions: oscillations and synchrony in top-down processing. Nat. Rev. Neurosci. 2, 704–716. doi: 10.1038/35094565, 11584308

[ref21] Flinker A. Chang E. F. Kirsch H. E. Barbaro N. M. Crone N. E. Knight R. T. (2010). Single-trial speech suppression of auditory cortex activity in humans. J. Neurosci. 30, 16643–16650. doi: 10.1523/JNEUROSCI.1809-10.2010, 21148003 PMC3010242

[ref22] Freeman J. A. Nicholson C. (1975). Experimental optimization of current source-density technique for anuran cerebellum. J. Neurophysiol. 38, 369–382. doi: 10.1152/jn.1975.38.2.369, 165272

[ref23] Fries P. (2005). A mechanism for cognitive dynamics: neuronal communication through neuronal coherence. Trends Cogn. Sci. 9, 474–480. doi: 10.1016/j.tics.2005.08.011, 16150631

[ref24] Fu K.-M. G. Shah A. S. O’Connell M. N. McGinnis T. Eckholdt H. Lakatos P. . (2004). Timing and laminar profile of eye-position effects on auditory responses in primate auditory cortex. J. Neurophysiol. 92, 3522–3531. doi: 10.1152/jn.01228.2003, 15282263

[ref25] Gegenfurtner K. R. (2016). The interaction between vision and eye movements †. Perception 45, 1333–1357. doi: 10.1177/0301006616657097, 27383394

[ref26] Ghazanfar A. A. Schroeder C. E. (2006). Is neocortex essentially multisensory? Trends Cogn. Sci. 10, 278–285. doi: 10.1016/j.tics.2006.04.008, 16713325

[ref27] Giehl J. Noury N. Siegel M. (2021). Dissociating harmonic and non-harmonic phase-amplitude coupling in the human brain. NeuroImage 227:117648. doi: 10.1016/j.neuroimage.2020.117648, 33338621 PMC7896041

[ref28] Gilbert C. D. Li W. (2013). Top-down influences on visual processing. Nat. Rev. Neurosci. 14, 350–363. doi: 10.1038/nrn3476, 23595013 PMC3864796

[ref29] Groh J. M. Trause A. S. Underhill A. M. Clark K. R. Inati S. (2001). Eye position influences auditory responses in primate inferior colliculus. Neuron 29, 509–518. doi: 10.1016/s0896-6273(01)00222-7, 11239439

[ref30] Haegens S. Zion Golumbic E. (2018). Rhythmic facilitation of sensory processing: a critical review. Neurosci. Biobehav. Rev. 86, 150–165. doi: 10.1016/j.neubiorev.2017.12.002, 29223770

[ref31] Helfrich R. F. (2018). The rhythmic nature of visual perception. J. Neurophysiol. 119, 1251–1253. doi: 10.1152/jn.00810.2017, 29357470

[ref32] Helfrich R. F. Knight R. T. (2016). Oscillatory dynamics of prefrontal cognitive control. Trends Cogn. Sci. 20, 916–930. doi: 10.1016/j.tics.2016.09.007, 27743685 PMC5127407

[ref33] Hoffman K. L. Dragan M. C. Leonard T. K. Micheli C. Montefusco-Siegmund R. Valiante T. A. (2013). Saccades during visual exploration align hippocampal 3-8 Hz rhythms in human and non-human primates. Front. Syst. Neurosci. 7:43. doi: 10.3389/fnsys.2013.00043, 24009562 PMC3757337

[ref34] Hubel D. H. Wiesel T. N. (1962). Receptive fields, binocular interaction and functional architecture in the cat’s visual cortex. J. Physiol. 160, 106–154. doi: 10.1113/jphysiol.1962.sp006837, 14449617 PMC1359523

[ref35] Ito J. Maldonado P. Grün S. (2013). Cross-frequency interaction of the eye-movement related LFP signals in V1 of freely viewing monkeys. Front. Syst. Neurosci. 7:1. doi: 10.3389/fnsys.2013.00001, 23420631 PMC3572441

[ref36] Ito J. Maldonado P. Singer W. Grün S. (2011). Saccade-related modulations of neuronal excitability support synchrony of visually elicited spikes. Cereb. Cortex 21, 2482–2497. doi: 10.1093/cercor/bhr020, 21459839 PMC3183421

[ref37] Jones E. G. (1998). Viewpoint: the core and matrix of thalamic organization. Neuroscience 85, 331–345. doi: 10.1016/s0306-4522(97)00581-2, 9622234

[ref38] Jones E. G. (2001). The thalamic matrix and thalamocortical synchrony. Trends Neurosci. 24, 595–601. doi: 10.1016/s0166-2236(00)01922-6, 11576674

[ref39] Jutras M. J. Fries P. Buffalo E. A. (2013). Oscillatory activity in the monkey hippocampus during visual exploration and memory formation. Proc. Natl. Acad. Sci. USA 110, 13144–13149. doi: 10.1073/pnas.1302351110, 23878251 PMC3740906

[ref40] Kajikawa Y. Schroeder C. E. (2011). How local is the local field potential? Neuron 72, 847–858. doi: 10.1016/j.neuron.2011.09.029, 22153379 PMC3240862

[ref41] Kajikawa Y. Schroeder C. E. (2015). Generation of field potentials and modulation of their dynamics through volume integration of cortical activity. J. Neurophysiol. 113, 339–351. doi: 10.1152/jn.00914.2013, 25274348 PMC4294577

[ref42] Kayser C. Logothetis N. K. (2007). Do early sensory cortices integrate cross-modal information? Brain Struct. Funct. 212, 121–132. doi: 10.1007/s00429-007-0154-0, 17717687

[ref43] Kayser C. Petkov C. I. Logothetis N. K. (2008). Visual modulation of neurons in auditory cortex. Cereb. Cortex 18, 1560–1574. doi: 10.1093/cercor/bhm187, 18180245

[ref44] Lakatos P. Barczak A. Neymotin S. A. McGinnis T. Ross D. Javitt D. C. . (2016). Global dynamics of selective attention and its lapses in primary auditory cortex. Nat. Neurosci. 19, 1707–1717. doi: 10.1038/nn.4386, 27618311 PMC5127770

[ref45] Lakatos P. Chen C. M. O’Connell M. N. Mills A. Schroeder C. E. (2007). Neuronal oscillations and multisensory interaction in primary auditory cortex. Neuron 53, 279–292. doi: 10.1016/j.neuron.2006.12.011, 17224408 PMC3717319

[ref46] Lakatos P. Karmos G. Mehta A. D. Ulbert I. Schroeder C. E. (2008). Entrainment of neuronal oscillations as a mechanism of attentional selection. Science 320, 110–113. doi: 10.1126/science.1154735, 18388295

[ref47] Lakatos P. Musacchia G. O’Connel M. N. Falchier A. Y. Javitt D. C. Schroeder C. E. (2013). The spectrotemporal filter mechanism of auditory selective attention. Neuron 77, 750–761. doi: 10.1016/j.neuron.2012.11.034, 23439126 PMC3583016

[ref48] Lakatos P. O’Connell M. N. Barczak A. McGinnis T. Neymotin S. Schroeder C. E. . (2020). The thalamocortical circuit of auditory mismatch negativity. Biol. Psychiatry 87, 770–780. doi: 10.1016/j.biopsych.2019.10.029, 31924325 PMC7103554

[ref49] Lakatos P. O’Connell M. N. Barczak A. Mills A. Javitt D. C. Schroeder C. E. (2009). The leading sense: supramodal control of neurophysiological context by attention. Neuron 64, 419–430. doi: 10.1016/j.neuron.2009.10.014, 19914189 PMC2909660

[ref50] Lakatos P. Pincze Z. Fu K.-M. G. Javitt D. C. Karmos G. Schroeder C. E. (2005a). Timing of pure tone and noise-evoked responses in macaque auditory cortex. Neuroreport 16, 933–937. doi: 10.1097/00001756-200506210-00011, 15931064

[ref51] Lakatos P. Shah A. S. Knuth K. H. Ulbert I. Karmos G. Schroeder C. E. (2005b). An oscillatory hierarchy controlling neuronal excitability and stimulus processing in the auditory cortex. J. Neurophysiol. 94, 1904–1911. doi: 10.1152/jn.00263.2005, 15901760

[ref52] Lee D. Malpeli J. G. (1998). Effects of saccades on the activity of neurons in the cat lateral geniculate nucleus. J. Neurophysiol. 79, 922–936. doi: 10.1152/jn.1998.79.2.922, 9463453

[ref53] Legatt A. D. Arezzo J. Vaughan H. G. (1980). Averaged multiple unit activity as an estimate of phasic changes in local neuronal activity: effects of volume-conducted potentials. J. Neurosci. Methods 2, 203–217. doi: 10.1016/0165-0270(80)90061-8, 6771471

[ref54] Leszczynski M. Bickel S. Nentwich M. Russ B. E. Parra L. Lakatos P. . (2023). Saccadic modulation of neural excitability in auditory areas of the neocortex. Curr. Biol. 33, 1185–1195.e6. doi: 10.1016/j.cub.2023.02.018, 36863343 PMC10424710

[ref55] Leszczynski M. Espinal E. Smith E. Schevon C. Sheth S. Schroeder C. E. (2025). Eye movements organize excitability state, information coding and network connectivity in the human hippocampus. Prog. Neurobiol. 252:102812. doi: 10.1016/j.pneurobio.2025.102812, 40816703

[ref56] Lohse M. Zimmer-Harwood P. Dahmen J. C. King A. J. (2022). Integration of somatosensory and motor-related information in the auditory system. Front. Neurosci. 16:1010211. doi: 10.3389/fnins.2022.1010211, 36330342 PMC9622781

[ref57] Mackey C. A. O’Connell M. N. Hackett T. A. Schroeder C. E. Kajikawa Y. (2024). Laminar organization of visual responses in core and parabelt auditory cortex. Cereb. Cortex 34:bhae373. doi: 10.1093/cercor/bhae373, 39300609 PMC11412770

[ref58] Maldonado P. Babul C. Singer W. Rodriguez E. Berger D. Grün S. (2008). Synchronization of neuronal responses in primary visual cortex of monkeys viewing natural images. J. Neurophysiol. 100, 1523–1532. doi: 10.1152/jn.00076.2008, 18562559

[ref59] Mégevand P. Mercier M. R. Groppe D. M. Zion Golumbic E. Mesgarani N. Beauchamp M. S. . (2020). Crossmodal phase reset and evoked responses provide complementary mechanisms for the influence of visual speech in auditory cortex. J. Neurosci. 40, 8530–8542. doi: 10.1523/JNEUROSCI.0555-20.2020, 33023923 PMC7605423

[ref60] Melloni L. Schwiedrzik C. M. Rodriguez E. Singer W. (2009). (Micro)saccades, corollary activity and cortical oscillations. Trends Cogn. Sci. 13, 239–245. doi: 10.1016/j.tics.2009.03.007, 19428286

[ref61] Mendoza-Halliday D. Major A. J. Lee N. Lichtenfeld M. J. Carlson B. Mitchell B. . (2024). A ubiquitous spectrolaminar motif of local field potential power across the primate cortex. Nat. Neurosci. 27, 547–560. doi: 10.1038/s41593-023-01554-7, 38238431 PMC10917659

[ref62] Mercier M. R. Foxe J. J. Fiebelkorn I. C. Butler J. S. Schwartz T. H. Molholm S. (2013). Auditory-driven phase reset in visual cortex: human electrocorticography reveals mechanisms of early multisensory integration. NeuroImage 79, 19–29. doi: 10.1016/j.neuroimage.2013.04.060, 23624493 PMC3677511

[ref63] Merzenich M. M. Brugge J. F. (1973). Representation of the cochlear partition of the superior temporal plane of the macaque monkey. Brain Res. 50, 275–296. doi: 10.1016/0006-8993(73)90731-2, 4196192

[ref64] Min B.-K. Busch N. A. Debener S. Kranczioch C. Hanslmayr S. Engel A. K. . (2007). The best of both worlds: phase-reset of human EEG alpha activity and additive power contribute to ERP generation. Int. J. Psychophysiol. 65, 58–68. doi: 10.1016/j.ijpsycho.2007.03.002, 17428561

[ref65] Mitzdorf U. (1985). Current source-density method and application in cat cerebral cortex: investigation of evoked potentials and EEG phenomena. Physiol. Rev. 65, 37–100. doi: 10.1152/physrev.1985.65.1.37, 3880898

[ref66] Mountcastle V. B. (1957). Modality and topographic properties of single neurons of cat’s somatic sensory cortex. J. Neurophysiol. 20, 408–434. doi: 10.1152/jn.1957.20.4.408, 13439410

[ref67] Naue N. Rach S. Strüber D. Huster R. J. Zaehle T. Körner U. . (2011). Auditory event-related response in visual cortex modulates subsequent visual responses in humans. J. Neurosci. 31, 7729–7736. doi: 10.1523/JNEUROSCI.1076-11.2011, 21613485 PMC6633117

[ref68] Nicholson C. Freeman J. A. (1975). Theory of current source-density analysis and determination of conductivity tensor for anuran cerebellum. J. Neurophysiol. 38, 356–368. doi: 10.1152/jn.1975.38.2.356, 805215

[ref69] Noesselt T. Tyll S. Boehler C. N. Budinger E. Heinze H.-J. Driver J. (2010). Sound-induced enhancement of low-intensity vision: multisensory influences on human sensory-specific cortices and thalamic bodies relate to perceptual enhancement of visual detection sensitivity. J. Neurosci. 30, 13609–13623. doi: 10.1523/JNEUROSCI.4524-09.2010, 20943902 PMC2958511

[ref70] O’Connell M. N. Barczak A. McGinnis T. Mackin K. Mowery T. Schroeder C. E. . (2020). The role of motor and environmental visual rhythms in structuring auditory cortical excitability. iScience 23:101374. doi: 10.1016/j.isci.2020.101374 32738615 PMC7394914

[ref71] O’Connell M. N. Barczak A. Ross D. McGinnis T. Schroeder C. E. Lakatos P. (2015). Multi-scale entrainment of coupled neuronal oscillations in primary auditory cortex. Front. Hum. Neurosci. 9:655. doi: 10.3389/fnhum.2015.00655 26696866 PMC4673342

[ref72] O’Connell M. N. Barczak A. Schroeder C. E. Lakatos P. (2014). Layer specific sharpening of frequency tuning by selective attention in primary auditory cortex. J. Neurosci. 34, 16496–16508. doi: 10.1523/JNEUROSCI.2055-14.2014, 25471586 PMC4252556

[ref73] O’Connell M. N. Falchier A. McGinnis T. Schroeder C. E. Lakatos P. (2011). Dual mechanism of neuronal ensemble inhibition in primary auditory cortex. Neuron 69, 805–817. doi: 10.1016/j.neuron.2011.01.012, 21338888 PMC3052772

[ref74] Obleser J. Kayser C. (2019). Neural entrainment and attentional selection in the listening brain. Trends Cogn. Sci. 23, 913–926. doi: 10.1016/j.tics.2019.08.004, 31606386

[ref75] Palva J. M. Palva S. Kaila K. (2005). Phase synchrony among neuronal oscillations in the human cortex. J. Neurosci. 25, 3962–3972. doi: 10.1523/JNEUROSCI.4250-04.2005, 15829648 PMC6724920

[ref76] Plass J. Ahn E. Towle V. L. Stacey W. C. Wasade V. S. Tao J. . (2019). Joint encoding of auditory timing and location in visual cortex. J. Cogn. Neurosci. 31, 1002–1017. doi: 10.1162/jocn_a_01399, 30912728 PMC6551606

[ref77] Popescu M. Otsuka A. Ioannides A. A. (2004). Dynamics of brain activity in motor and frontal cortical areas during music listening: a magnetoencephalographic study. NeuroImage 21, 1622–1638. doi: 10.1016/j.neuroimage.2003.11.002, 15050586

[ref78] Ramcharan E. J. Gnadt J. W. Sherman S. M. (2001). The effects of saccadic eye movements on the activity of geniculate relay neurons in the monkey. Vis. Neurosci. 18, 253–258. doi: 10.1017/s0952523801182106, 11417800

[ref79] Rauschecker J. P. Tian B. Pons T. Mishkin M. (1997). Serial and parallel processing in rhesus monkey auditory cortex. J. Comp. Neurol. 382, 89–103. doi: 10.1002/(SICI)1096-9861(19970526)382:1<89::AID-CNE6>3.0.CO;2-G, 9136813

[ref80] Rayner K. (1998). Eye movements in reading and information processing: 20 years of research. Psychol. Bull. 124, 372–422. doi: 10.1037/0033-2909.124.3.372, 9849112

[ref81] Reppas J. B. Usrey W. M. Reid R. C. (2002). Saccadic eye movements modulate visual responses in the lateral geniculate nucleus. Neuron 35, 961–974. doi: 10.1016/s0896-6273(02)00823-1, 12372289

[ref82] Robinson D. L. McClurkin J. W. Kertzman C. (1990). Orbital position and eye movement influences on visual responses in the pulvinar nuclei of the behaving macaque. Exp. Brain Res. 82, 235–246. doi: 10.1007/BF00231243, 2286229

[ref83] Romei V. Gross J. Thut G. (2012). Sounds reset rhythms of visual cortex and corresponding human visual perception. Curr. Biol. 22, 807–813. doi: 10.1016/j.cub.2012.03.025, 22503499 PMC3368263

[ref9002] Saleem K. S. Logothetis N. K. (2012). A Combined MRI and Histology Atlas of the Rhesus Monkey Brain in Stereotaxic Coordinates (2nd ed.) Elsevier S & T. p. 402.

[ref84] Schmehl M. N. Herche J. L. Groh J. M. (2025). Visually evoked activity and variable modulation of auditory responses in the macaque inferior colliculus. J. Neurophysiol. 133, 1456–1467. doi: 10.1152/jn.00529.2024, 40111400 PMC12131782

[ref85] Schneider L. Dominguez-Vargas A.-U. Gibson L. Wilke M. Kagan I. (2023). Visual, delay, and oculomotor timing and tuning in macaque dorsal pulvinar during instructed and free choice memory saccades. Cereb. Cortex 33, 10877–10900. doi: 10.1093/cercor/bhad333, 37724430

[ref86] Schneider D. M. Nelson A. Mooney R. (2014). A synaptic and circuit basis for corollary discharge in the auditory cortex. Nature 513, 189–194. doi: 10.1038/nature13724, 25162524 PMC4248668

[ref87] Schroeder C. E. Lakatos P. (2009). Low-frequency neuronal oscillations as instruments of sensory selection. Trends Neurosci. 32, 9–18. doi: 10.1016/j.tins.2008.09.012, 19012975 PMC2990947

[ref88] Schroeder C. E. Mehta A. D. Givre S. J. (1998). A spatiotemporal profile of visual system activation revealed by current source density analysis in the awake macaque. Cereb. Cortex 8, 575–592. doi: 10.1093/cercor/8.7.575, 9823479

[ref89] Schroeder C. E. Lindsley R. W. Specht C. Marcovici A. Smiley J. F. Javitt D. C. (2001). Somatosensory input to auditory association cortex in the macaque monkey. J. Neurophysiol. 85, 1322–1327. doi: 10.1152/jn.2001.85.3.1322, 11248001

[ref90] Schroeder C. E. Wilson D. A. Radman T. Scharfman H. Lakatos P. (2010). Dynamics of active sensing and perceptual selection. Curr. Opin. Neurobiol. 20, 172–176. doi: 10.1016/j.conb.2010.02.010, 20307966 PMC2963579

[ref91] Shah A. S. Bressler S. L. Knuth K. H. Ding M. Mehta A. D. Ulbert I. . (2004). Neural dynamics and the fundamental mechanisms of event-related brain potentials. Cereb. Cortex 14, 476–483. doi: 10.1093/cercor/bhh009, 15054063

[ref9003] Sherman S. M. Guillery R. W. (1998). On the actions that one nerve cell can have on another: distinguishing “drivers” from “modulators”. Proceedings of the National Academy of Sciences of the United States of America. 95, 7121–7126. doi: 10.1073/pnas.95.12.7121, 9618549 PMC22761

[ref92] Sherman S. M. Guillery R. W. (2002). The role of the thalamus in the flow of information to the cortex. Philos. Trans. R. Soc. Lond. Ser. B Biol. Sci. 357, 1695–1708. doi: 10.1098/rstb.2002.1161, 12626004 PMC1693087

[ref93] Sherman S. M. Guillery R. W. (2011). Distinct functions for direct and transthalamic corticocortical connections. J. Neurophysiol. 106, 1068–1077. doi: 10.1152/jn.00429.2011, 21676936

[ref94] Steinschneider M. Reser D. Schroeder C. E. Arezzo J. C. (1995). Tonotopic organization of responses reflecting stop consonant place of articulation in primary auditory cortex (A1) of the monkey. Brain Res. 674, 147–152. doi: 10.1016/0006-8993(95)00008-e, 7773684

[ref95] Sylvester R. Haynes J.-D. Rees G. (2005). Saccades differentially modulate human LGN and V1 responses in the presence and absence of visual stimulation. Curr. Biol. 15, 37–41. doi: 10.1016/j.cub.2004.12.061, 15649362

[ref96] Thorne J. D. De Vos M. Viola F. C. Debener S. (2011). Cross-modal phase reset predicts auditory task performance in humans. J. Neurosci. 31, 3853–3861. doi: 10.1523/JNEUROSCI.6176-10.2011, 21389240 PMC6622791

[ref97] Vinck M. Uran C. Spyropoulos G. Onorato I. Broggini A. C. Schneider M. . (2023). Principles of large-scale neural interactions. Neuron 111, 987–1002. doi: 10.1016/j.neuron.2023.03.015, 37023720

[ref98] Vittek A.-L. Juan C. Gaillard C. Mercier M. Girard P. Ben Hamed S. . (2025). Frequency coding of multisensory integration in the local field potentials of the medial pulvinar. Eur. J. Neurosci. 62:e70230. doi: 10.1111/ejn.70230, 40888183 PMC12400260

[ref99] Vittek A.-L. Juan C. Nowak L. G. Girard P. Cappe C. (2023). Multisensory integration in neurons of the medial pulvinar of macaque monkey. Cereb. Cortex 33, 4202–4215. doi: 10.1093/cercor/bhac337, 36068947 PMC10110443

[ref100] Wallace M. T. Stein B. E. (1994). Cross-modal synthesis in the midbrain depends on input from cortex. J. Neurophysiol. 71, 429–432. doi: 10.1152/jn.1994.71.1.429, 8158240

