# Supplementary material for: Spectrotemporal signatures of driving- and modulatory circuits across cortical and subcortical networks
Source: Front Hum Neurosci. 2026 Aug 12;20:1868940. doi: 10.3389/fnhum.2026.1868940 (PMC13506714; doi:10.3389/fnhum.2026.1868940)
Supplement: Supplementary file 1 [file Data_Sheet_1.pdf]

Supplementary Materials:

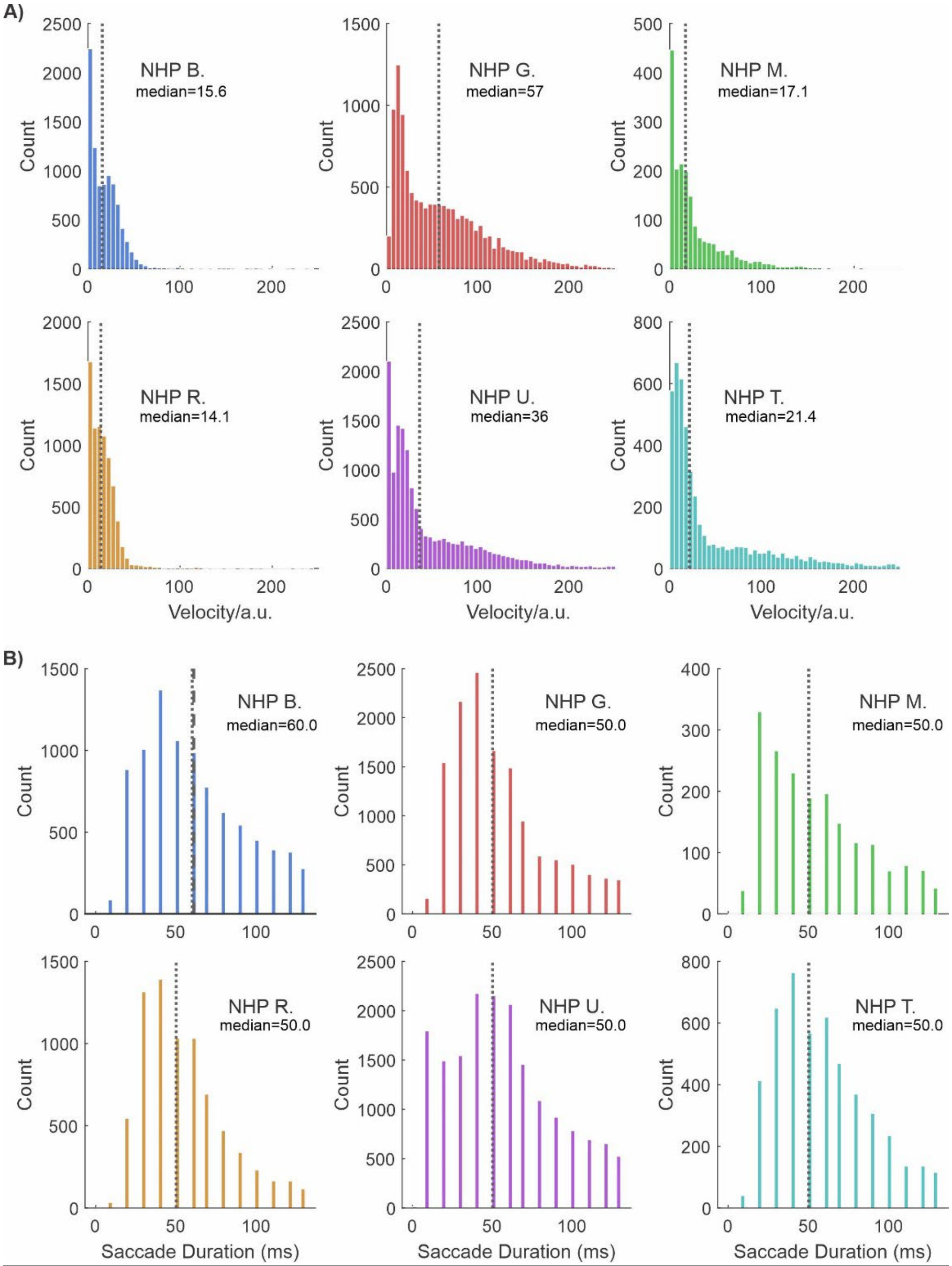

Supplementary Figure 1. Saccade velocity and duration distributions across NHPs. A) Histograms of saccade velocity (in arbitrary units) for each of the 6 NHPs. Dashed vertical lines indicate the median velocity for each animal. **B)** Histograms of saccade duration (ms) for each NHP. Dashed vertical lines indicate the median duration. Saccade durations ranged from 8 to 130ms, with median durations of 50-60ms across animals.

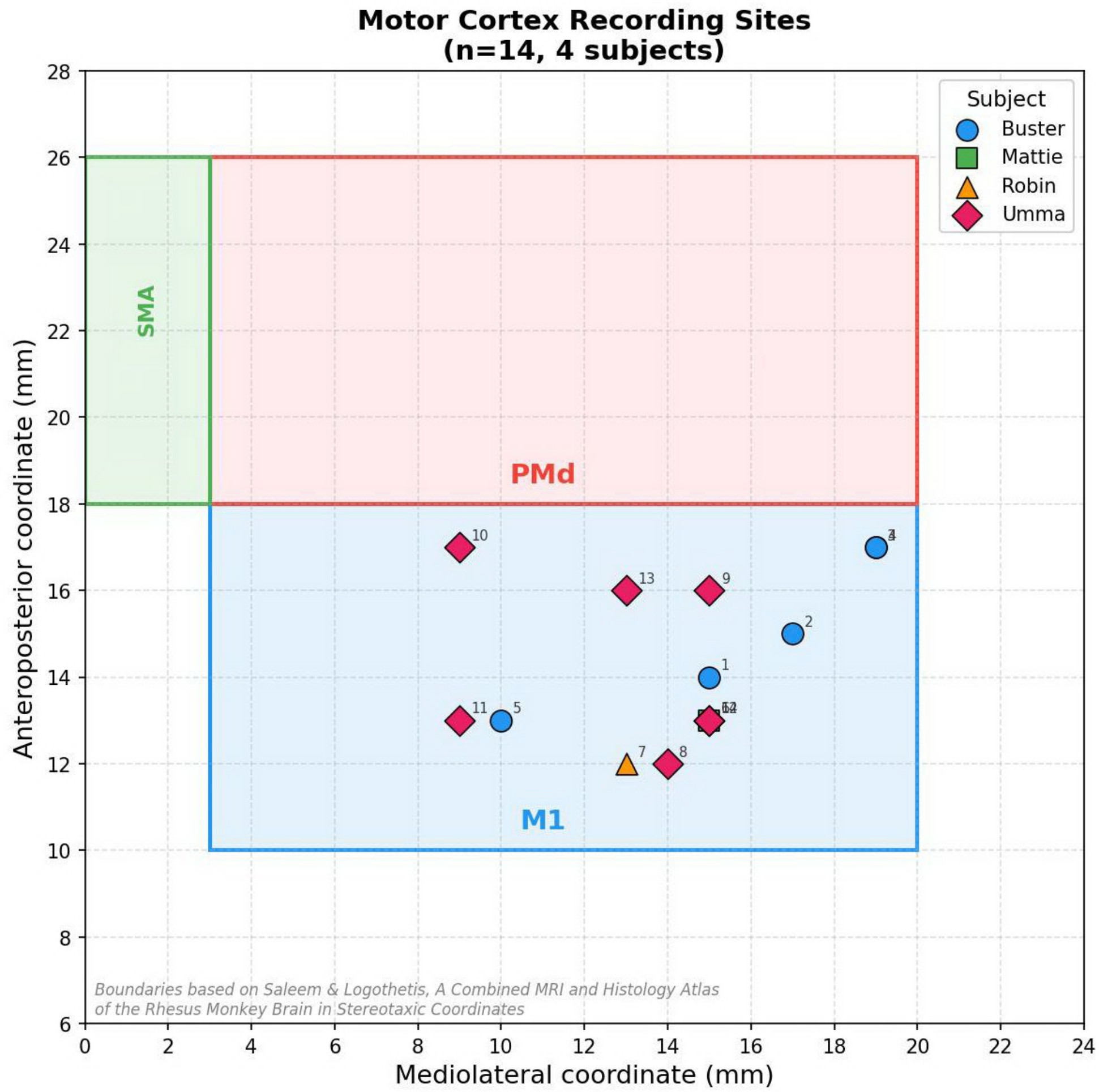

Supplementary Figure 2. Motor cortex recording site localization. Stereotaxic coordinates of all 14 motor cortex recording sites plotted in anteroposterior (AP) vs. mediolateral (ML) space across 4 NHPs. Different symbols denote different subjects. Shaded regions indicate anatomical boundaries for primary motor cortex (M1, blue), dorsal premotor cortex (PMd, red), and supplementary motor area (SMA, green) as defined by Saleem and Logothetis (A Combined MRI and Histology Atlas of the Rhesus Monkey Brain in Stereotaxic Coordinates). Coordinates are given in mm.

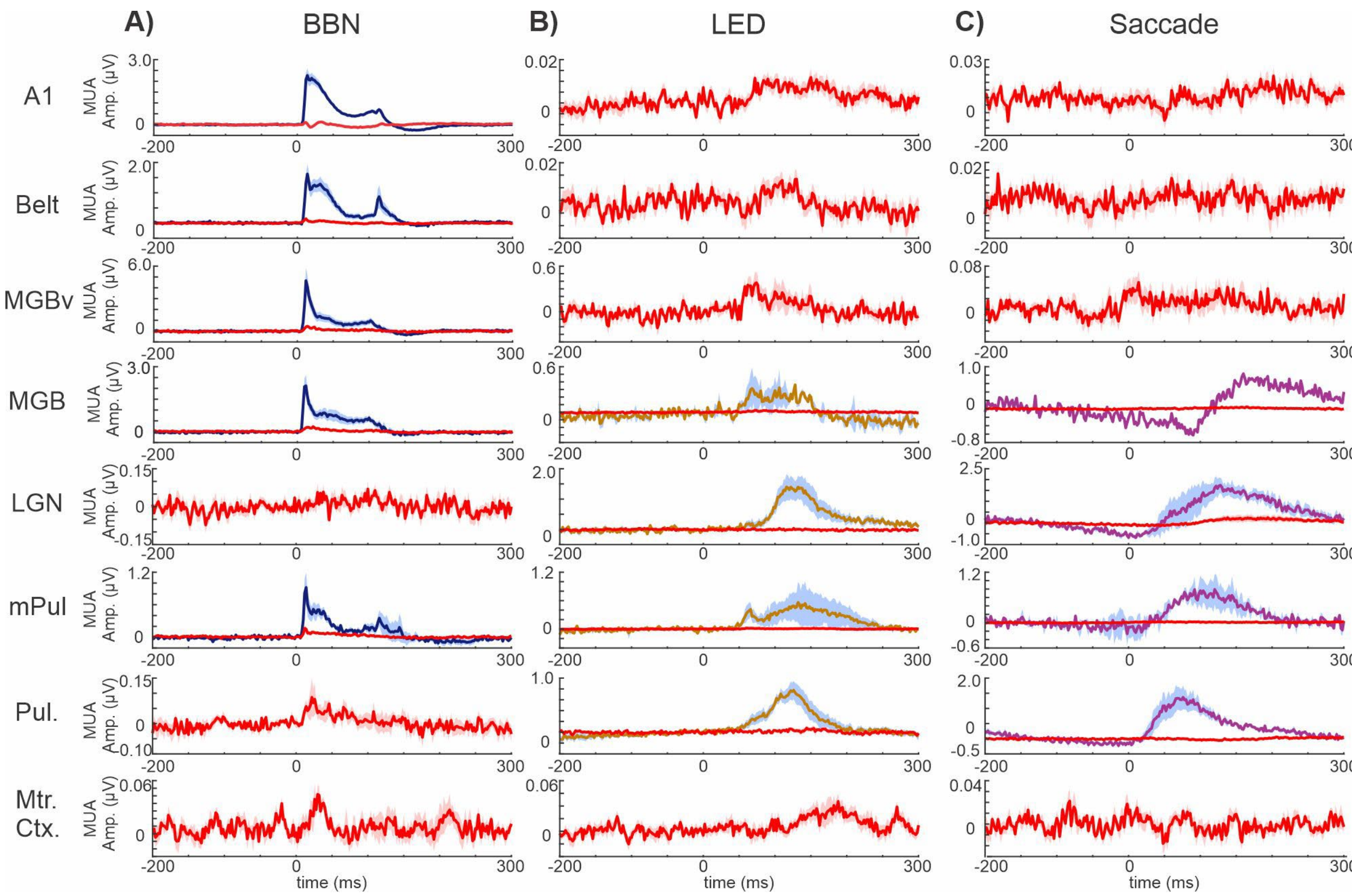

Supplementary Figure 3. Population mean MUA responses by brain area and stimulus condition. A) Population mean BBN-related MUA responses (± SEM, shaded). **B)** Population mean LED-related MUA responses (± SEM, shaded). **C)** Population mean saccade-related MUA responses (± SEM, shaded). Significant evoked MUA responses are shown in color (dark blue: BBN; mustard: LED; purple: Saccade); non-significant responses are shown in red. Note that the y-axis scale varies across panels, as non-significant response amplitudes are substantially smaller than significant ones. Time 0 denotes stimulus onset for A and B, and saccade onset for C.

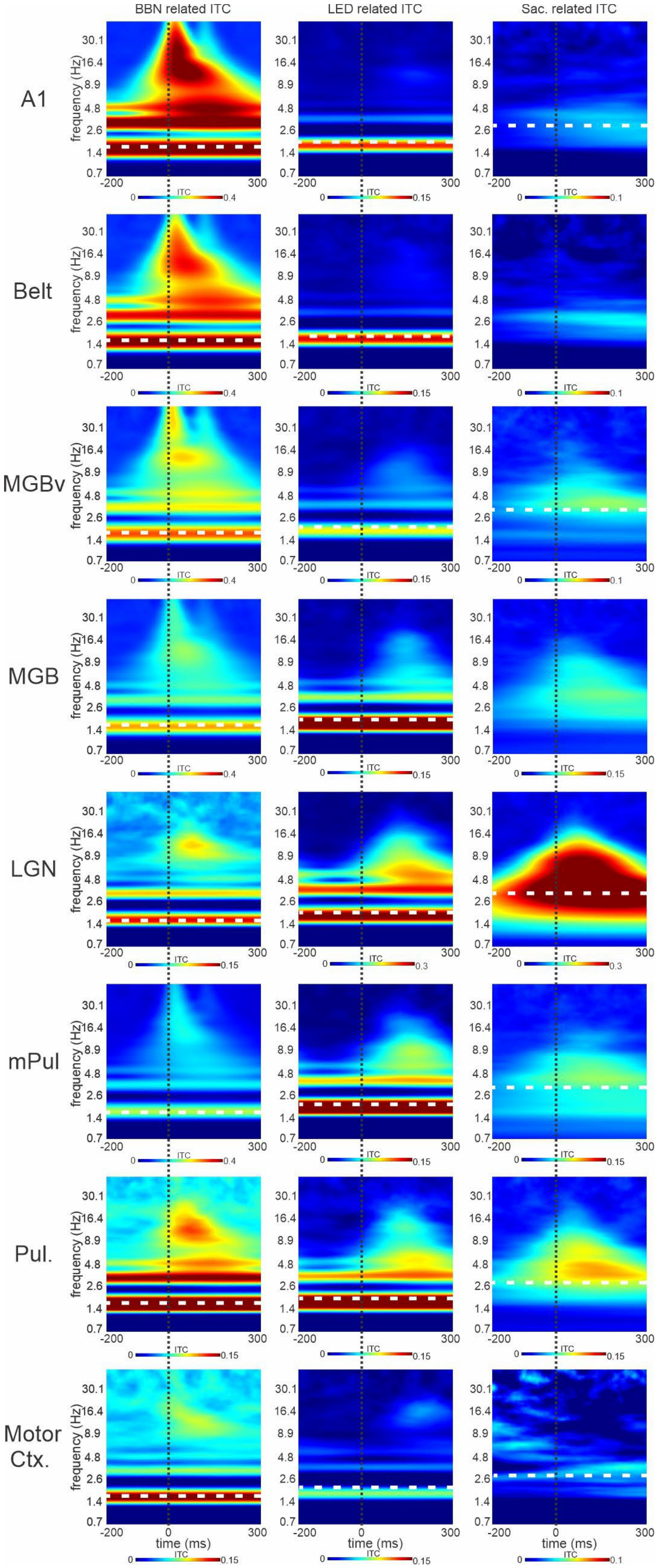

Supplementary Figure 4. Population-mean spectrotemporal ITC heatmaps by brain area and stimulus condition. Each panel shows the population-mean inter-trial coherence (ITC) across time and frequency for each brain area (rows) and stimulus condition. White dashed horizontal lines indicate the stimulus or event rates (BBN: 1.6Hz; LED: 1.8Hz; saccade: median rate). Black vertical dashed lines mark time 0ms (stimulus or saccade onset). Color scales differ across areas and conditions to optimize visualization of the ITC range within each panel.

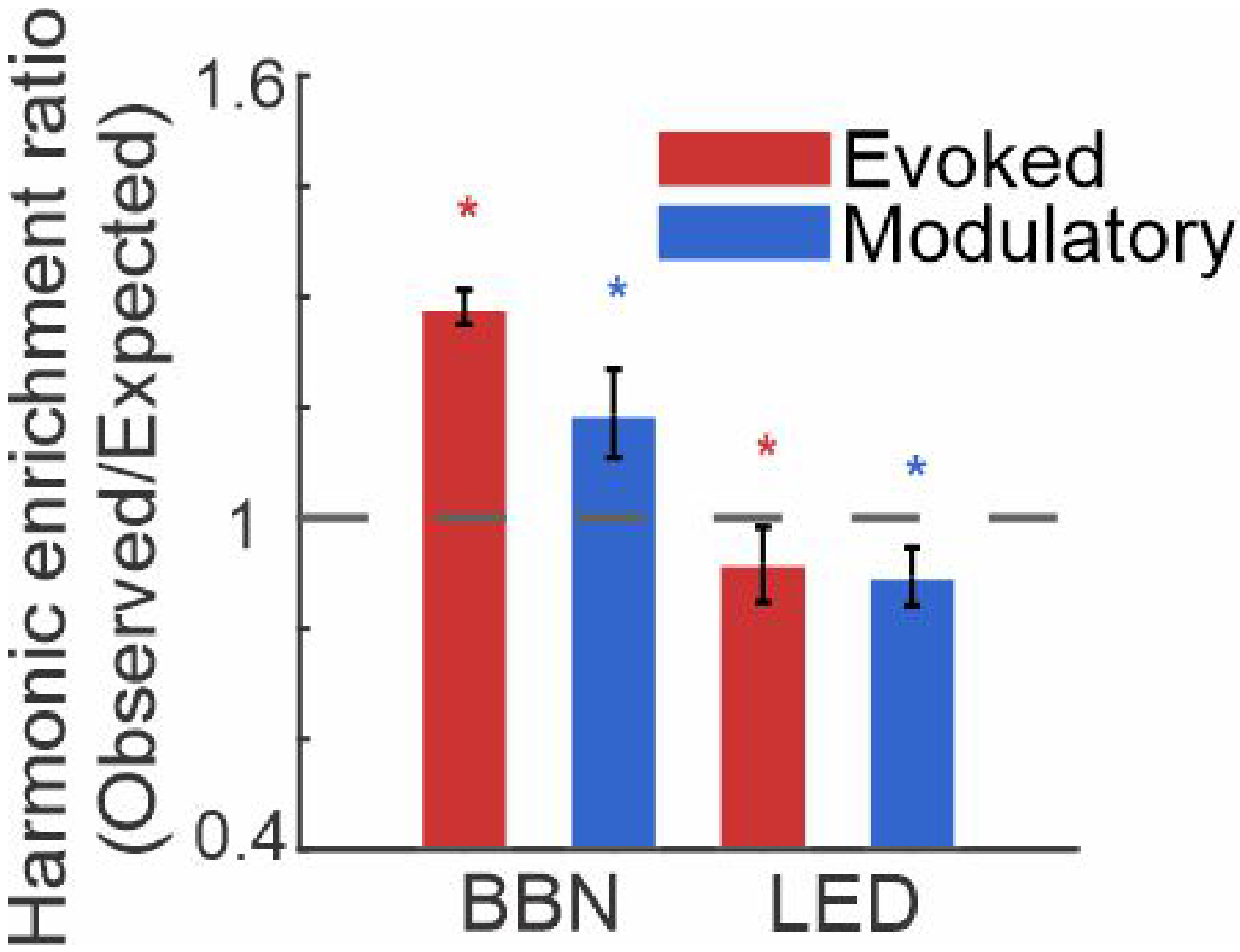

Supplementary Figure 5: Bars show the ratio of observed to expected proportion of significant ITC peaks falling at harmonic frequency bins, for evoked (red) and modulatory (blue) responses collapsed across all 8 brain areas. A ratio of 1.0 (dashed line) indicates peaks fall at harmonic frequencies at the rate expected by chance (BBN: 27.6% of resolvable bins; LED: 26.7%). Ratios above 1.0 indicate greater harmonic concentration than chance; ratios below 1.0 indicate peaks preferentially avoid harmonic frequencies. Error bars = 95% bootstrap CI (n=1000). Asterisks indicate ratios significantly different from chance. Analysis restricted to resolvable frequency range (BBN: <26.66Hz; LED: <30.11Hz).

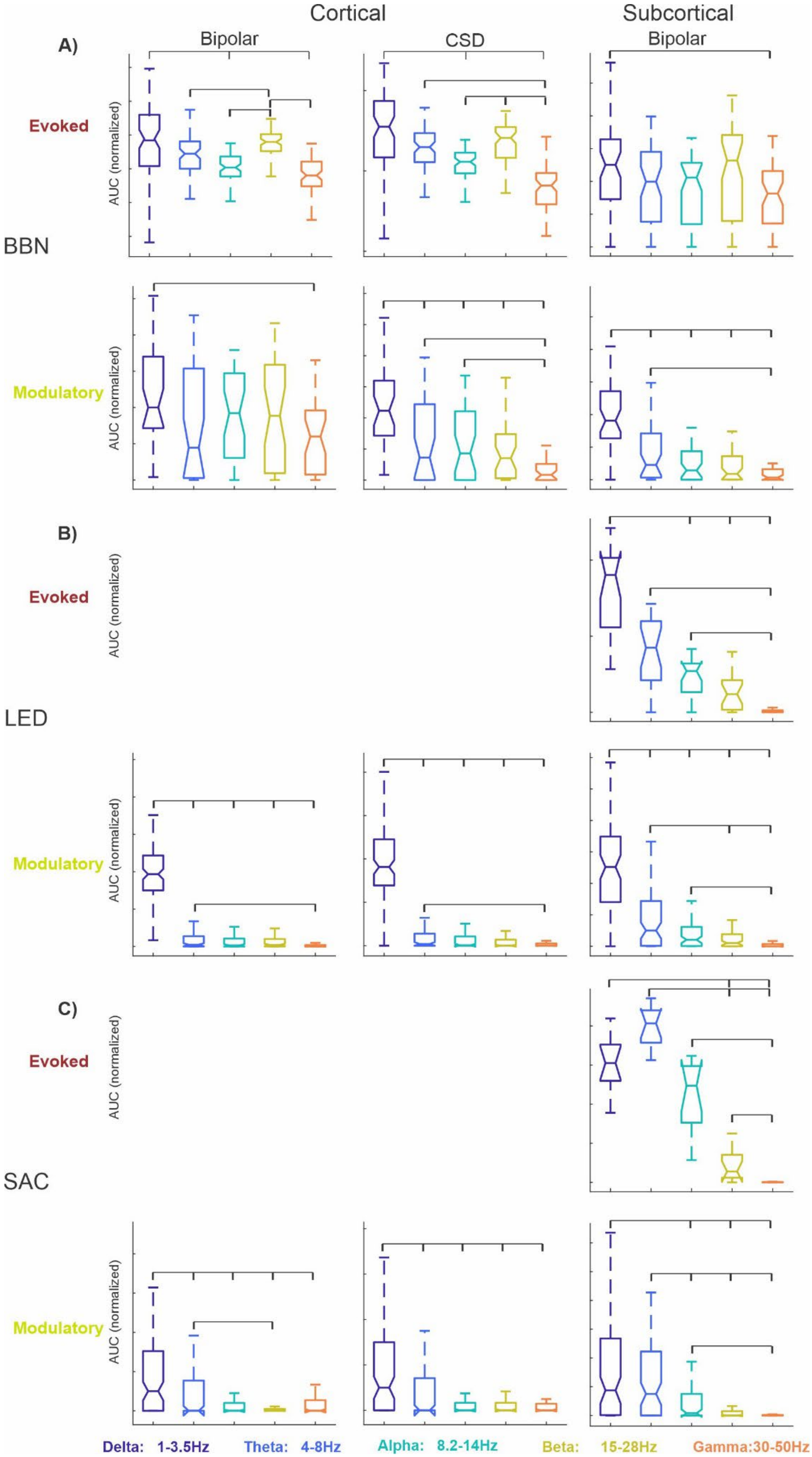

Supplementary Figure 6. Statistical comparison of frequency band-limited AUCs of significant ITC peak distributions by spatial derivation. (A–C) Boxplots show pooled area under the curve (AUC) values for the frequency distribution of significant ITC peaks across five canonical frequency bands, separated by response type (evoked, top; modulatory, bottom), stimulus condition, and spatial derivation (cortical bipolar, left; cortical CSD, middle; subcortical bipolar, right). AUCs were computed from sites exhibiting evoked or modulatory responses and pooled across all brain regions within each derivation type. Brackets indicate significant differences between frequency bands (Kruskal–Wallis test with Bonferroni correction, $p < 0.01$). Empty panels indicate conditions where no evoked or modulatory responses were observed for that derivation type. A) BBN-related responses. B) LED-related responses. C) Saccade-related responses.
